# Beyond Barriers: Active Packaging Strategies for Sustainable Food Protection

**DOI:** 10.3390/polym18111399

**Published:** 2026-06-04

**Authors:** Elisabetta Maffioli, Marco Ruggeri, Carmela Tommasino, Barbara Vigani, Silvia Rossi, Giuseppina Sandri

**Affiliations:** Department of Drug Sciences, University of Pavia, Via Torquato Taramelli 12, 27100 Pavia, Italy; elisabetta.maffioli01@universitadipavia.it (E.M.);

**Keywords:** FLW, active packaging, global sustainability, shelf life, food preservation, packaging nanomaterials

## Abstract

Food loss and waste—FLW—represent a critical global challenge, primarily across postharvest handling, storage, and distribution. Shelf life limitations—arising from microbial activity and proliferation, physicochemical degradation, and environmental interactions—are major contributors to these losses. Intrinsic factors such as pH, water activity, nutrient composition, and biological structure interact with extrinsic conditions including temperature, humidity, gaseous atmosphere, and light exposure, ultimately leading to quality deterioration and consumer rejection. A comprehensive insight into these mechanisms is essential to improve preservation strategies and reduce FLW. This review critically examines the determinants of food shelf life and highlights the strategic role of innovative packaging technologies in mitigating degradation pathways. Particular emphasis is placed on active packaging systems, including commonly studied technologies such as oxygen and ethylene scavengers, carbon dioxide emitters and absorbers, moisture regulators, antimicrobial- and antioxidant-releasing materials, and flavor and odor control systems. Their mechanisms of action, material design, performance factors, and practical limitations are discussed. Innovative packaging technologies actively modulate spoilage, extend shelf life, and preserve both sensory and nutritional quality, moving beyond conventional passive barriers. When combined with optimized supply chains and sustainable materials, these systems can strengthen food system stability and advance global sustainability goals.

## 1. Introduction

Food crises occur when hunger, food insecurity, and malnutrition increase sharply or persist at local, national, or global levels due to disruptions in food supply or demand [1]. The current global food crisis is affecting millions of people worldwide. According to the World Food Programme, at least 319 million people across 67 countries are expected to face acute food insecurity in 2025. This represents a substantial increase compared with pre-pandemic levels, driven by wars and geopolitical conflicts (19 countries/territories; 147.4 M people), natural disasters and extreme weather events (16 countries/territories; 87.5 M people), and economic shocks (12 countries/territories; 29.8 M people), which have further increased the costs of fuel, food, and fertilizers. The burden is particularly severe in South Asia and the Pacific, Central and Southern Africa, West Africa and the Sahel, and East Africa, where conflict, climate-related disruptions, and economic instability are the predominant drivers of food crises. These pressures are often compounded by large-scale population displacement and acute malnutrition among vulnerable groups, especially children under 5 years of age and pregnant or breastfeeding women, further exacerbating food insecurity at regional and global scales [2].

Moreover, despite the ever-growing variety of sales formats, the rapid expansion of the online food market, and the adoption of a circular economy that pushes for reuse, recycling, or recovery, the tendency is to waste [3,4].

**Figure 1:**
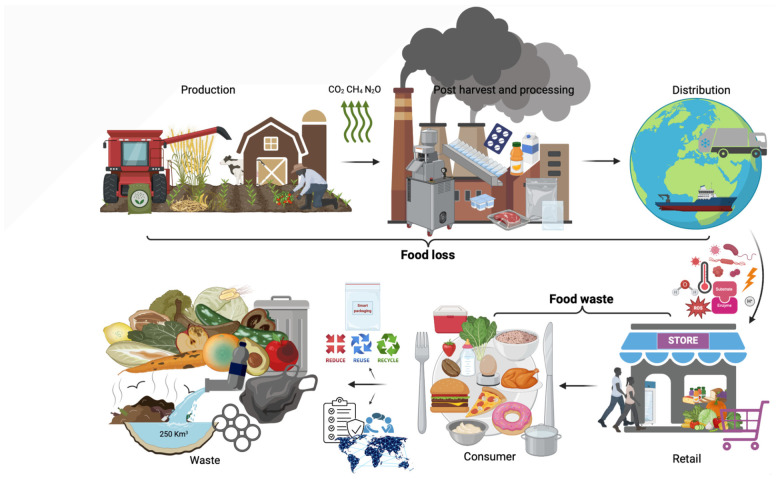
FLW along the food supply chain and their environmental impacts. Food loss occurs during agricultural production, post-harvest handling, processing, and distribution, whereas food waste primarily occurs at retail and consumer stages. These losses contribute to greenhouse gas emissions, resource depletion, and waste generation, highlighting the need for sustainable management strategies based on waste prevention, reduction, reuse, and recycling. Created in BioRender. Ruggeri, M. (2026) https://BioRender.com/mn7tdrj (accessed on 28 May 2026).

Global food loss is caused by inadequate food production and deficient supply systems or malfunctioning of institutional and policy frameworks. Food loss takes place at the earlier stages of the food supply chain and can be a consequence of poor storage facilities, inadequate temperature-controlled supply chains, poor food handling, insufficient infrastructure and transportation practices, deficient packaging design, or lack of efficient marketing systems [3,5]. In low- and middle-income countries (North Africa, Central and Western Asia, Latin America and the Caribbean, and South and Southeastern Asia), food loss occurs predominantly at the agricultural production stage (approximately 20–30%) and during postharvest handling and storage (around 30–40%), mainly due to inadequate harvesting practices, insufficient storage infrastructure, limited cold-chain systems, and inefficient transportation [5]. Recent FAO reports indicate that similar postharvest challenges persist in Central and Southern Asia, including India and Bangladesh, and in Latin America and the Caribbean, which continue to represent a significant challenge across the agrifood system [6]. Food waste is referred to the removal from food supply chain of edible food still fit for human consumption. Food loss and waste (FLW) is increasing to 1.3 billion tons per year, aggravating the food crises [3]. Indeed, approximately a third of the food in the world is wasted every year—for a monetary value estimated at roughly US$ 1 trillion [7]—throughout the entire supply chain, together with the resources employed for its production, processing, and distribution (Figure 1) [3]. Food waste can be a consequence of lack of adequate purchase and consumption planning, absence of awareness and education, inadequate quality, poor environmental conditions during display, and scarce communication in supply chains [3]. In medium- and high-income countries (Europe, USA Canada, Australia and New Zealand, and especially industrialized Asia) about 40% of food waste occurs at the consumption level [8]. FLW occurs during production, postharvest, and processing stages or at the retail and consumer levels [3,9]. What emerges is that, both in low-developed countries and in high-developed countries, around 130 Mtons of FLW occurs at the postharvest handling and storage levels or the processing and packaging stages. FLW has many adverse environmental, economic, financial, and natural resource impacts [3], with effects on the environment directly related to the disposal of unwanted food or indirectly related to the energy inputs employed in food production [10]. This contributes to climate change, with generation of anthropogenic greenhouse gases (GHG) such as carbon oxide (CO_2_), methane (CH_4_), and nitrous oxide (N_2_O) [11,12,13]. On a 20-year timescale, CH_4_ has 84 times greater global warming potential than CO_2_ [14], and N_2_O can trap over 250 times more heat than CO_2_ [12]. FLW accounts for a third of global GHG emissions, with the release of 4.4 gigatons of CO_2_ equivalents from edible food waste that has not been recycled [3,11]. The analysis by Ivanovich et al. (2023) [12] showed that >80% of future warming from food consumption will be from meat, rice, and dairy products: precisely, high-methane food groups. The food supply chain consumes considerable amounts of water, accounting for 250 km^3^ of blue water in 2019 (1.5 times the volume of the Dead Sea) [4,15]. Food production also requires the generation and use of nitrogen fertilizers, as well as the use of diesel ploughing, harvesting, and drying [9]. Food manufacturing causes the degradation of natural ecosystems and biodiversity loss [16,17]. The socioeconomic impact of FLW is of severe concern, considering that, although the production of food in the world is adequate, nearly a billion people worldwide suffer from hunger and malnutrition [16,18]. In fact, part of the population has access to sufficient or surplus food, but low-income subpopulations do not have the opportunity to acquire quality foods or do not have enough food at all [18,19].

Therefore, the UN announced the Sustainable Development Goals (SDGs) agreed on in September 2015. Goal 12.3 aims to “halve per capita global food waste at the retail and consumer levels and reduce food losses along the food production and supply chains, including post-harvest losses, by 2030”, and goal 12.5 aims to “substantially reduce waste generation through prevention, reduction, recycling and reuse by 2030” [20]. Reducing FLW not only represents one of the leading global strategies towards meeting a sustainable food future, but also can alleviate poverty, reduce food insecurity, and decrease the pressure on ecosystems, climate, and water [9]. Reducing FLW can increase the efficiency of the food supply chain, lowering costs for producers and prices for consumers [9,17,21]. Notably, the amount of FLW could alleviate around 12% of undernourishment worldwide [8]. Cutting FLW in half (from 24% to 12%) by the year 2050 could reduce the global need for about 1314 trillion kilocalories (kcal) per year of food—roughly 22% of the 6000 trillion kcal per year gap between the food that is available today and that needed in 2050 [17]. For these reasons, strategies to reduce FLW are necessary to manage government investments, coordinate food supply chain stages, establish appropriate policymaking addressing market access, and develop efficient distribution systems. Finally, coordination and communication at the manufacturer/customer interface, together with better consciousness of FLW and appropriate dietary guidelines, are essential [3,8,22,23].

## 2. Factors Affecting Food Shelf Life

Food loss at the handling and storage stages accounts for the largest portion of total FLW [17]. Adequate transportation infrastructure and vehicles and timely transportation from storage facilities to retail by careful forecasting of demand are essential. These factors could guarantee the quality standards of fresh products such as fish, meat, vegetables, and fruits that can easily deteriorate, assure optimal storage on the retail shelf, and maintain highly perishable products in proper conditions [8,24].

**Figure 2:**
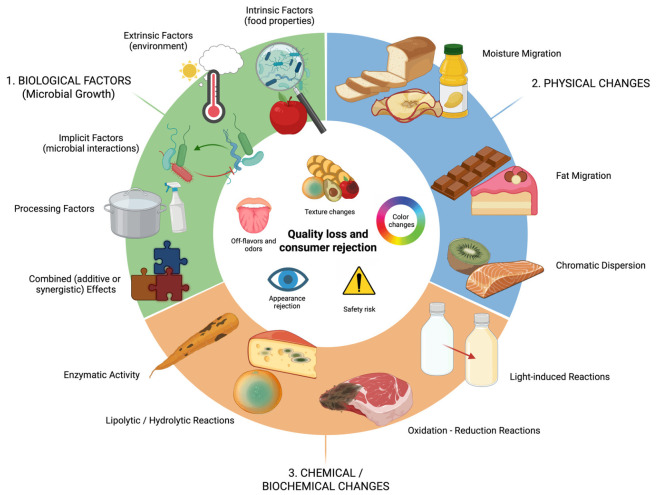
Illustrative summary of the main biological, physical, and chemical factors affecting food shelf life and contributing to quality deterioration, including sensory, nutritional, and safety losses. Created in BioRender. Ruggeri, M. (2026) https://BioRender.com/7f3wa0y (accessed on 27 May 2026).

The factors affecting food shelf life include microbial growth, physical, chemical, or biochemical changes in the food itself, or changes resulting from the activity of microorganisms or parasites [24,25]. Figure 2 provides an overview of the main biological, physical, and biochemical factors affecting food shelf life.

### 2.1. Microbial Growth

Factors affecting the growth of microorganisms in food can be:

- intrinsic parameters such as hydrogen ion concentration (pH), amount of water available for biological functions, oxidation–reduction potential, nutrient content, presence of antimicrobial constituents, competitive microflora, or biological structures that prevent microbial entry [26,27]
- extrinsic factors include storage temperature, relative humidity, gaseous atmosphere, exposure to light, or activity of other microorganisms [27,28]
- implicit factors of food comprise metabiosis, symbiosis, commensalism, antagonism, synergism, predation, or amensalism [27]
- food processing factors include physical treatments such as heating, cooling, and drying, use of chemicals, or contamination [27]
- additive or synergistic interaction of the above-described factors can affect microbial growth [29].

### 2.2. Physical Changes

Physical deteriorative changes in food are mainly related to moisture migration, but also to migration of fat in multicomponent foods or to chromatic dispersion [30]. Moisture migration in a multi-domain food is influenced by gradients in water activity (thermodynamics) and rate of diffusion (dynamics of mass transfer), driving forces that lead to a (re)distribution phenomena from one region of a food component to another [30,31,32]. Practical examples include the loss of crisp texture in breakfast cereals, wafers, biscuits, and snack products; progressive leathering of bread-like products; caking in products with high levels of soluble sugars, minerals, or protein hydrolysates (e.g., milk powders, instant coffee, and dehydrated fruit juices), resulting in particle agglomeration (also known as wet granulation); and moisture-induced crystallization in spray-dried milk powders, ice cream, hard candies, soft cookies, and baked goods. These phenomena can reduce powder flowability, promote granular texture development in ice cream, increase cookie firmness, and impair the visual and sensory quality of hard candies, which may become opaque, lose gloss, and develop a sandy or grainy mouthfeel, ultimately reducing consumer acceptability [33]. Physical factors limiting the shelf life of food systems therefore include crystallization, stickiness, and undesirable texture transitions, such as crispy-to-soft, hard-to-rubbery, or leathery changes. Physical changes occurring in packaging systems, potentially coupled with chemical reactions, may also promote migrative phenomena that impair food shelf life [29].

### 2.3. Chemical Changes

Chemical deteriorative changes result from reactions within the food, reactions between the food and the surrounding environment species, or reactions after its processing [34,35]. Enzymatic activity, lipolytic/hydrolytic reactions, redox reactions, and exposure to light are the main determinants of chemical changes. These mechanisms are responsible for undesirable flavors, brown pigmentation, loss of protein digestibility/solubility, color changes, and vitamin loss [34]. A temperature-controlled regime is essential to control the interactions among the food, packaging material, and storage duration [36]. An optimal temperature preserves sensory and nutritional qualities, prevents microbial contamination, spoilage, and the risk of food poisoning, reduces the rate of chemical reactions, controls crystallization characteristics, and stabilizes emulsion systems [37,38].

Quality decreases with time, occurring between harvest and consumption. The cumulative effect of the changes can reach a point at which the subjective consumer evaluation determines goods rejection. Rejection is based on a complex of quality attributes that include sensory perceptions and expectations such as taste, texture, color, and appearance [28,38]. To prevent this occurrence, manufacturers can regulate different intrinsic and extrinsic factors which impact on shelf life [39]. These include raw material selection and quality, product formulation and assembly, the processing environment, processing and preservation techniques, packaging materials, storage and distribution, and consumer practice [40,41]. In particular, the research community addressed its attention to the development of novel processing methods and packaging design [42,43].

## 3. Active Packaging Technologies

Food packaging is an essential element meant to contain, protect, and preserve foods, and hence to retain the desired quality throughout the shelf life [44,45]. From a socioeconomic point of view, food packaging can lower the cost of many foods, can reduce energy consumption, and can provide important on-pack information to the customer [46]. When designing a food packaging system, there are some aspects that should be considered, such as energy supplies, environmental impact and sustainability, material selection, costs, and customer perception [46,47]. Among the most promising and constantly growing technologies are active packaging systems [48].

According to EC/450/2009, “active materials and articles” mean materials and articles that are intended to extend the shelf life and maintain or improve the condition of packaged food. They are designed to deliberately incorporate components that would release or absorb substances into or from the packaged food or the environment surrounding the food. The reasons to consider active packaging for food and beverage derive from the typical constrains of any package selection. These matters include economic costs, engineering limitations, environmental impacts, side effects of traditional packaging, and time-dependent degradative processes [49].

The most remarkable active packaging solutions comprise oxygen and ethylene scavenging, carbon dioxide scavenging or release, moisture regulators, antimicrobial packaging, antioxidant release, and release or adsorption of flavors and odors [49,50]. Other active packaging systems comprise self-heating, self-venting, and microwavable active packaging [51].

Among the various strategies developed for active packaging, the most widely used are gas-control systems—including oxygen (O_2_), ethylene (C_2_H_4_), and carbon dioxide (CO_2_) regulation—moisture control systems, antimicrobial packaging, antioxidant packaging, flavor/odor control systems, and multifunctional systems. Table 1 summarizes the main technologies, including their mechanisms of action, representative active agents, applications, and main limitations.

### 3.1. Gas-Control Systems

#### 3.1.1. Oxygen-Scavenging Technology

**Figure 3:**
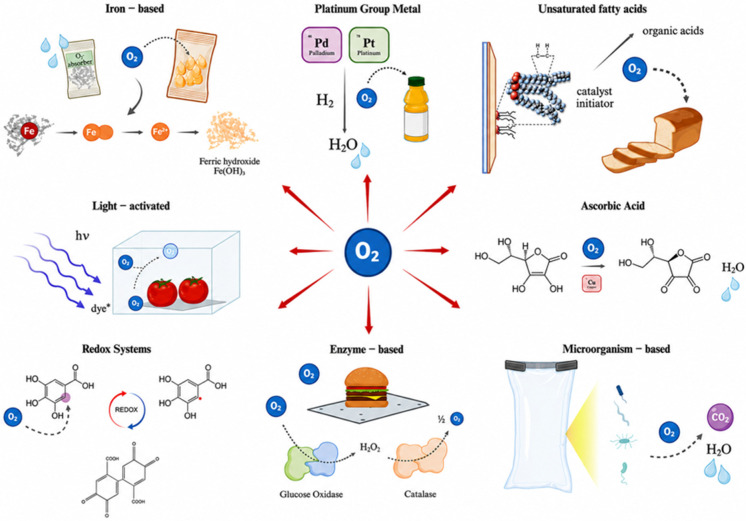
Schematic overview of the principal O_2_ scavenger systems used in active food packaging. The figure illustrates iron-based (oxidation of Fe^2+^ to Fe^3+^) (top, left), platinum group metal-catalyzed (catalytic reduction of O_2_ to H_2_O in the presence of H_2_) (top, middle), unsaturated fatty acid-based (autoxidation/polymerization reactions consuming O_2_) (top, right), light-activated (photoinduced oxygen consumption via dyes/photosensitizers) (middle, left), ascorbic acid-based (oxidation of ascorbic acid to dehydroascorbic acid) (middle, right), redox system-based (electron-transfer oxidation–reduction reactions) (bottom, left), enzyme-mediated (enzymatic conversion of O_2_, e.g., glucose oxidase/catalase reactions) (bottom, middle), and microorganism-based (microbial respiration consuming O_2_ and producing CO_2_ and H_2_O) (bottom, right) O_2_ scavengers. Created in BioRender. Ruggeri, M. (2026). https://BioRender.com/hv6cqhp (accessed on 27 May 2026).

Nowadays, the most employed active packaging technology for foods is oxygen scavenging. O_2_ levels depend on metabolic activity or permeation through the packaging system [50,52]. In modified atmosphere packaging (MAP), high levels of O_2_ serve to retain color, minimize drip losses, and inhibit anaerobic microorganisms in poultry and fresh red meat [53]. O_2_ above 30% helps minimize both the growth of anaerobic pathogens and drip losses in white fish [54]. Elevated levels of O_2_ have also inhibiting effects on enzymatic browning in both intact and fresh-cut fruits and vegetables, may control the formation of volatile compounds and reduce off flavors, and cause a significant reduction in the growth rate of various microorganisms associated with vegetables [55]. However, O_2_ present in food packages causes food spoilage due to oxidation of fats or vitamins or promotes the growth of microorganisms such as aerobic bacteria, yeasts, and molds [56]. The interaction between food and oxygen results in loss of freshness, change of color, smell, or taste, and decrease in nutritive values [52,57]. In this regard, different strategies have been developed to eliminate oxygen from the packaged food environment. O_2_-sensitive foods can be successfully packaged in MAP or vacuum packaging [58]. However, these technologies cannot always remove the small quantity of residual O_2_ (accounting for up to 0.5–2 vol. %), limiting their application to highly perishable foods [56,57]. Oxygen scavengers can absorb residual O_2_ after packaging (reducing oxygen levels in the headspace to less than 0.01%), minimizing the quality changes of O_2_-sensitive foods [50,57]. O_2_-scavenging technologies work by oxidizing metallic or non–metallic substrates. As represented in Figure 3 and listed in Table 2, the most-used oxygen-scavenging systems use iron-based, ascorbic acid-based, light-activated, enzyme-based, unsaturated fatty acid-based, redox-based, phenolic-based, and microorganism-based scavengers [58].

##### Metal-Based Scavengers

The most widely used oxygen scavenging systems are iron-based scavengers, typically consisting of powder combined with catalytic components to accelerate oxidation. The reaction that takes place is the oxidation of the scavenger in presence of moisture or a Lewis acid like FeCl_3_ or AlCl_3_ [57]. The main reactions involved in the mechanism of oxidation can be expressed in Equations (1)–(4) [61]:

Fe → Fe^2+^ + 2 e^−^ (anodic reaction) (1)

½ O_2_ + H_2_O + 2 e^−^ → 2 OH^−^ (cathodic reaction) (2)

Fe^2+^ + 2 OH → Fe(OH)_2_ (formation of ferrous hydroxide) (3)

Fe(OH)_2_ + ¼ O_2_ + ½ H_2_O → Fe(OH)_3_ (oxidation to ferric hydroxide) (4)

Overall reaction:

4Fe + 3O_2_ + 6H_2_O → 4Fe(OH)_3_

Certain scavengers incorporate water in their carrier matrix, enabling self-activation upon contact with oxygen. In contrast, others depend on product moisture for activation and are thus applicable only to moist products. Commercially available self-activated oxygen-scavenging systems are typically composed of an obverse printed polyethylene terephthalate (PET) layer laminated to paper and subsequently to low-density polyethylene (LDPE) and/or linear low-density polyethylene (LLDPE). The active scavenging component—iron powder—is blended with moist zeolite (or another suitable carrier material) that serves as a water reservoir. In contrast, moisture-dependent sachet systems feature one side made from a low-moisture-barrier material such as Tyvek^®^ [a perforated high-density polyethylene (HDPE)] that allows moisture from the packaged product to permeate. This moisture activates the scavenging reaction upon exposure to oxygen [59,78]. A novel oxygen scavenger based on iron nanoparticles placed inside a sachet was also developed, and its ability to inhibit lipid oxidation was demonstrated with roasted sunflower seeds and walnuts. Compared to the conventional oxygen scavenger, the amount of O_2_ absorbed by the nanosized scavenger increased from 95.3 ± 4.8 mL to 134.1 ± 2.9 mL. Furthermore, the peroxide value (a measure of the concentration of peroxides and hydroperoxides formed during the early stages of lipid oxidation) of roasted sunflower seeds after 120 days of storage at 25 °C was 19.82 mEq O_2_·kg^−1^ oil. This value complies with the Chinese national hygienic standard, which sets a maximum peroxide value of 39.4 mEq O_2_·kg^−1^ oil for roasted nuts [61]. Oxygen-scavenging polyolefin nanocomposite films containing an iron modified kaolinite were seen to play a dual oxygen-fighting role: (i) the more tortuous gas permeation path that the kaolinite platelets impose onto the polymeric matrix; and (ii) the iron contained in the kaolinite traps that reacts with the molecular oxygen, causing the active reduction of gas pressure through the composite. Humidity and temperature are key factors in the kinetics of oxygen depletion of active iron-based nanocomposites; indeed, active films were able to uptake between 2.4 O_2_·g^−1^ and 4.3 mL O_2_·g^−1^ at 24 °C and 100% RH, but the activity decreased almost by half at 5 °C and 50% RH [79].

The disadvantages of iron-based scavenging systems include the environmental dependence of the package’s oxygen-scavenging capacity and reaction rate, potential metal contamination, accidental setting off of in-line metal detectors, of the inability to use microwave heating, and the temperature-dependent nature of iron oxidation [80]. Although iron nanoparticles incorporated into oxygen-scavenging systems can significantly enhance oxygen absorption, concerns remain regarding their potential migration from the packaging material into the food matrix and the consequent consumer exposure. Currently, limited information is available on the extent of nanoparticle migration and the associated dietary exposure from nano-enabled food packaging systems, while regulatory frameworks governing their use in food-contact materials remain only partially harmonized. Iron nanoparticles are generally considered among the more biocompatible nanomaterials; however, their potential toxicity depends on physicochemical characteristics such as particle size, surface properties, concentration, shape, and structural composition. Therefore, comprehensive migration, toxicological, and risk assessment studies are necessary before large-scale commercial implementation [81,82].

Metal-based (non-iron) systems, particularly those based on platinum group metals (PGMs), are employed, where noble metals catalyze the reaction between hydrogen and oxygen to form water [57,58]. The catalytic oxidation of hydrogen with oxygen proceeds according to Equation (5) [83]:

(5) $$H_{2}+½O_{2}\overset{Pd/Pt}{\to }H_{2}O$$

Platinum group metal-based scavenging systems display high efficiency in catalyzing the conversion of hydrogen and oxygen into water [57,84].

Non-metallic scavengers include those that use organic reducing agents such as ascorbic acid, photosensitive dies, enzymes (such as glucose oxidase and alcohol oxidase), unsaturated fatty acids, rice extract, or immobilized yeast on a solid substrate [50,56,57].

##### Reducing Agent-Based Scavengers

The ascorbic acid reaction as oxygen scavenger is based on ascorbate oxidation to dehydroascorbic acid coupled to Cu^2+^ reduction to Cu^+^. The newly formed Cu^+^ complexes with the O_2_, forming superoxide anionic radical and restoring Cu^2+^. In the presence of copper, the radical generates O_2_ and H_2_O_2_. The copper–ascorbate complex quickly reduces the H_2_O_2_ to H_2_O without OH^−^ formation, according to Equation (6) [58]:

AA + ½ O_2_ → DHAA + H_2_O, (6)

where AA is ascorbic acid and DHAA is dehydroascorbic acid.

Lee et al. (2018) [64] developed an optimum formulation of nonferrous oxygen scavenger particles (NFOSPs), consisting of 100 g of activated carbon and 160 g of sodium *L*-ascorbate with 1 mL of water, to enhance the preservation of raw meatloaves. In their study, oxygen concentrations were measured during storage, and the NFOSP160/1 sachet maintained a continuously decreasing oxygen concentration, reaching below 1% by day 4. The oxygen scavenger was also effective in retrding lipid oxidation and controlling microbial growth. Janjarasskul et al. (2011) [85] developed an ascorbic acid-containing whey protein film with effective scavenging activity against O_2_ initially present in the package headspace, as well as oxygen permeating through the packaging wall over time. Edible oxygen-scavenger films offer the advantages of avoiding accidental consumption and addressing the non-recyclability issues associated with conventional oxygen scavenger systems.

A major limitation of ascorbic acid-based oxygen scavengers, including those used with iron powder, is that they require the presence of moisture to become active.

##### Light-Based Scavengers

Photosensitive dye oxidation is the main light-activated oxygen scavenger [73]. When the package is irradiated with light of the appropriate wavelength (near-infrared, visible, or ultraviolet radiation), the dye activates O_2_ to its singlet state, accelerating the oxygen-removing reaction [50,57]. The photochemical reaction proceeds as follows in Equations (7)–(9) [73]:

photon + dye → dye* (photoexcitation)(7)

dye* + O_2_ → dye + O_2_* (energy transfer to oxygen) (8)

O_2_* + acceptor → acceptor peroxide (oxidation of the acceptor) (9)

Ramakanth et al. (2024) [67] developed an oxygen-scavenging material by combining plant-based natural rubber latex (NRL) with polyvinyl alcohol (PVA) to fabricate biodegradable films aimed at extending the shelf life of food products. The film containing a 2:1 ratio of NRL exhibited the highest scavenging capacity, achieving 1045 mL O_2_ per g and 95 mL O_2_ per g per day at 60 °C with 120 s of UV-C exposure. Owing to its high capacity and reaction rate, the oxygen concentration in the package dropped to zero within a few days. The scavenging reaction is activated by UV-C light, which generates singlet oxygen via radicals produced when the photo-initiator is exposed to UV-C. In NRL, the singlet oxygen attacks the allylic carbons of unsaturated bonds, consuming oxygen and thereby reducing its content inside the package. Increasing the UV-C exposure time accelerates singlet oxygen formation, enhancing oxygen scavenging. Additionally, the films demonstrated improved barrier properties against water vapor, as indicated by reduced water vapor transmission rates and increased crystallinity. Amberg-Schwab et al. (2023) [65] developed a novel UV-activated, transparent oxygen-scavenging coating based on inorganic–organic polymers (ORMOCER^®^) applied to aluminum foil and laminated with a polyethylene sealing layer. The functional mechanism of this newly developed system relies on the photo-initiated, metal-catalyzed oxidation of a cyclo-olefin moiety chemically bonded to a silicate backbone. This design enables activation of the scavenging process by UV irradiation while preventing the formation of low-molecular-weight oxidation products that could compromise the quality of packaged goods or pose potential toxicity concerns. The oxygen absorption capacity, measured at 23 °C and 0% relative humidity, was 242 ± 8 mg O_2_ per g of scavenger coating. When the coating layer was laminated using a two-component polyurethane adhesive, the oxygen absorption capacity remained high, at 223 ± 18 mg O_2_ per g of scavenger, indicating the feasibility of covering the scavenger layer with a functional barrier that can minimize migration of the photo-initiator into the packaged products.

Major limitations may be associated with the potential migration of residual photo-initiators from the scavenger layer into the packaged food, although this risk can be mitigated by incorporating a functional barrier layer between the scavenger system and the food product. Additional concerns relate to the exposure of sensitive food components to UV irradiation during activation of the oxygen scavenger. UV irradiation is well known to promote lipid and protein oxidation, cause vitamin degradation, induce color instability through protein denaturation, increase exposure of hydrophobic sites, increase myoglobin autoxidation, alter texture due to protein degradation, and generate undesirable flavor and odor compounds [86,87,88,89]. These detrimental effects may be mitigated by optimizing UV irradiation parameters, including dose, intensity, and exposure time, thereby supporting the development of a sustainable preservation technology that improves food safety and quality [90]. Furthermore, the oxygen-depleting activity of the scavenger during storage may help minimize subsequent oxidative deterioration by limiting oxygen availability within the package [87]. In commercial ORMOCER^®^-based coatings, UV activation is performed immediately before the packaging process, while the formation of low-molecular-weight oxidation products is minimized by combining hybrid polymer coatings with thin inorganic layers that form a dense crosslinked network. A similar technology was adopted commercially in the Cryovac^®^ OS1000 and OS2000 systems (Sealed Air, Charlotte, NC, USA) [65,91].

##### Enzyme-Based Scavengers

Glucose oxidase and catalase can be combined to react with a specific substrate to scavenge any incoming O_2_. Briefly, the glucose oxidase transfers two hydrogens from the -CHOH group of glucose (originally present in the food or in the scavenger formulation) to O_2_ with the formation of glucono-δ-lactone and H_2_O_2_. The lactone spontaneously reacts with water to form gluconic acid. The catalase breaks down the H_2_O_2_ to water and oxygen, decreasing the system efficiency. The common reaction pathway can be represented in Equations (10) and (11) [50,92]:

2 glucose + 2O_2_ + 2H_2_O → 2 gluconic acid + 2 H_2_O_2_ (glucose oxidation) (10)

2H_2_O_2_ → 2H_2_O + O_2_ (hydrogen peroxide decomposition) (11)

Overall reaction of the combined enzyme system (glucose oxidase and catalase):

2 glucose + O_2_ + 2H_2_O → 2 gluconic acid + 2H_2_O

Ge et al. (2012) [93] developed a novel food packaging material by immobilizing glucose oxidase within a poly(vinyl alcohol)/chitosan/tea extract electrospun nanofibrous membrane, which exhibited both antibacterial and oxygen-scavenging abilities. By combining glucose oxidase with chitosan and tea extract and immobilizing the complex on the electrospun membrane, the system effectively prevented potential adverse effects on human health that could arise from the direct addition of oxygen-scavenging agents. This approach broadened the scope of application by maintaining the original flavor and quality of the packaged food while simultaneously achieving antibacterial activity and oxygen control.

More enzymes that can be added to the scavenger formulation include laccase, ethanol oxidase, or oxalate oxidase, along with catalase [50,57,58,80]. Coupled enzyme systems are very sensitive to some parameters, such as changes in pH, salt content, temperature, and water activity, and they also require the addition of water, reducing the efficiency for low-water foodstuffs. Johansson et al. (2012) [94] investigated the oxygen-scavenging capacity of laccase immobilized within a polymeric substrate composed of lignosulfonate—a material consisting of a hydrophobic backbone of phenylpropane units with hydrophilic sulfonate and hydroxyl groups—embedded in latex-based or starch-based films and coatings applied to foil or board. Laccase is a copper-containing enzyme that catalyzes the one-electron oxidation of phenolic hydroxyl groups to phenoxy radicals, while molecular oxygen is reduced to water, as represented in Equation (12):

(12) $$4PhOH+O_{2}\;\overset{laccase}{\to }4PhO+2H_{2}O$$

The results indicated that laccase and lignosulfonate can function effectively as an oxygen-scavenging system for active packaging of high-moisture foods. After 6 days at 23 °C and 100% relative humidity, the oxygen content in airtight chambers decreased from 1.0% to 0.3% in the presence of a board coated with lignosulfonate and laccase, whereas the oxygen content remained unchanged in control samples without the enzyme [94].

##### Unsaturated Hydrocarbon-Based Scavengers

Among the unsaturated organic scavengers, the polyunsaturated fatty acids represent the main category. The main advantage of this kind of oxygen scavenger resides in the fact that it can be activated in absence of water, enabling its usage to package dry foods [57,58]. Oleic, linoleic, or linolenic acids, arachidonic acid, parinaric acid, dimer acid, or ricinoleic acid in the presence of catalysts, or initiators such as heat, light/ionizing radiation, and metal ions/metalloproteins lose a hydrogen atom and produce free radicals. The free radicals react with oxygen in the presence of a carrier substance that absorbs oxygen molecules [52,71]. Others ethylenic-unsaturated hydrocarbons used as oxygen scavengers include squalene or polybutadiene. The reaction proceeds via autoxidation of the oxidizable substrate, as shown in Equations (13)–(15) [57]:

RH + O_2_ → ROOH (autoxidation) (13)

ROOH + M^n+^ → RO + M^(n+1)+^ + OH^−^ (metal-catalyzed decomposition) (14)

ROOH + M^(n+1)+^ → ROO^−^ + M^n+^ + H^+^ (propagation) (15)

where M refers to a metal complex as catalyst and RH refers to the allylic carbon hydrogen bonds of a polymeric section of the unsaturated hydrocarbon.

The main limitation of this technology resides in the fact that, during the reaction between polyunsaturated molecules and oxygen, the generation of by-products such as organic acids, aldehydes, or ketones can affect the quality of the food [58,71]. Röcker et al. (2021) [72] investigated the oxygen-scavenging activity of modified calcium carbonate (MCC) particles loaded with oleic acid (OA) and linoleic acid (LA). For both LA and OA, a 20 wt. % loading on MCC carriers provided optimal surface area-to-volume ratios, facilitating oxygen exposure and reaction across numerous molecular sites. This resulted in oxygen scavenging rates of 12.2 ± 0.6 and 1.7 ± 0.2 mL O_2_·d^−1^·g^−1^ and maximum oxygen absorption capacities exceeding 195.6 ± 13.5 and 165.0 ± 2.0 mL·g^−1^, respectively. For both fatty acids, oxygen-scavenging activity decreased with increasing relative humidity (37–100% RH) but increased with higher temperatures (5–30 °C). Therefore, MCC loaded with unsaturated fatty acids (UFAs) appears particularly suitable for food products with low water activity that are stored under non-refrigerated conditions.

##### Redox-Based Scavengers

Among redox-based oxygen scavenger systems, quinones such as 2-methylanthraquinone or 2-ethylanthraquinone are considered particularly suitable. These compounds can be activated in absence of transition metal catalysts; instead, they may be triggered by light with a proper wavelength, heat, gamma radiation, corona discharge, or electron beam. The reducible organic compounds are excited to a higher energy state, which can gain or subtract an electron or a hydrogen atom. The reduced state that follows is reactive against oxygen to produce hydrogen peroxide, hydroperoxyl radical, or superoxide radical. These reactive intermediates are then absorbed by reactive compounds incorporated within the system, such as antioxidants [57,73]. The reaction proceeds via autoxidation of the oxidizable substrate, as shown in Equations (16)–(18) [73]:

RH → R^·^ (initiation) (16)

R^·^ + O_2_ → ROO^·^ (peroxyl radical formation) (17)

ROO^·^ + RH → ROOH + R^·^ (propagation) (18)

Overall reaction:

RH + O_2_ → ROOH,

where R is the reducible organic group.

##### Phenolic Compound-Based Scavengers

Also, natural phenolic compounds such as gallic acid (GA) act as free radical scavengers. GA absorbs large amounts of O_2_ under alkaline conditions, provided by combination with a base such as potassium carbonate (K_2_CO_3_), sodium carbonate (Na_2_CO_3_), or sodium hydroxide (NaOH) and a moisture supply. GA oxidizes with the formation of hydrogen peroxide, quinones, and semi-quinones, following Equations (19) and (20) [75,95]:

K_2_CO_3_ + H_2_O → 2 KOH + CO_2_ (activation step) (19)

GallicH + O_2_ → Gallic• + HO_2_• (scavenging step) (20)

HO_2_• + GallicH → Gallic• + H_2_O_2_

2 Gallic• → Gallic acid dimer

Gallic acid dimer + O_2_ → Quinone of dimer + 2 H_2_O_2_

Quinone of dimer + 2 O_2_ → Open-ring product of dimer

##### Microorganism-Based Scavengers

Microorganisms entrapped in a polymeric matrix can act as natural and biological oxygen scavengers [58]. Microorganisms remove oxygen via aerobic respiration, following Equation (21) [96]:

Organic substrate + O_2_^−^ → CO_2_ + H_2_O + energy (21)

Aerobic microorganisms such as *Kocuria varians* and *Pichia subpelliculosa* can work as active compounds to develop an environmentally friendly oxygen-scavenger film [77].

Also, endospore-forming bacteria can be used as active oxygen scavengers for plastic packaging materials [97].

A summary of the described oxygen scavenging technologies is presented in Table 3. The table includes information on the active material, polymer matrix, technology type, O_2_-scavenging capacity, O_2_ absorption rate, functional performance, storage conditions, applications, and corresponding references, where available.

Oxygen scavenger systems contribute to sustainability primarily by extending shelf life and reducing oxidative spoilage, thereby helping to mitigate food loss and waste across the supply chain. However, their sustainability should also be evaluated in terms of the packaging materials themselves. Conventional oxygen scavengers are commonly iron-based sachets, which, despite their effectiveness, introduce additional packaging components that may complicate waste management and recycling [104,105].

To address these limitations, increasing attention has been directed toward integrated oxygen-scavenging packaging systems, including polymer- and biopolymer-based films, coatings, and active bottle systems. In particular, non-iron scavengers based on natural compounds such as plant-derived polyphenols have emerged as promising alternatives due to their potential compatibility with biodegradable packaging matrices. Nevertheless, challenges related to cost, scalability, regulatory approval, and end-of-life management remain significant barriers to their broader implementation [74,106].

#### 3.1.2. Ethylene Scavengers and Adsorbers

Ethylene (C_2_H_4_) is a simple unsaturated hydrocarbon that function as a phytohormone and hence has numerous effects on growth, development, and storage life of many fruits, vegetables, and ornamental crops, like seed germination, cell elongation, flower development, senescence, defense against pathogens, and response to external stress factors, among others. In addition to the several beneficial effects on some foods—such us promoting uniform ripening—it can also negatively impact the quality and storage life of other products [107,108]. According to the ethylene production and cellular respiration in the stage of fruit ripening, fruits are broadly classified as climacteric and non-climacteric. Climacteric fruits, such as apple, mango, papaya, avocado, kiwifruit, banana, pear, blueberry, and broccoli, exhibit a distinct rise in both respiration rate and C_2_H_4_ production during ripening [109]. This negatively affects the shelf life of these products, causing excessive tissue softening/toughening, color changes, weight loss, sugar content alteration, and the development of characteristic aroma and flavor compounds [108]. In contrast, non-climacteric fruits, such as citrus fruits, pineapple, melon, peas, pepper, cacao, and cucumber, do not exhibit a pronounced increase in respiration, with C_2_H_4_ production remaining at basal levels [110]. In these fruits, C_2_H_4_ mainly acts as a senescence signal, promoting chlorophyl degradation and the appearance of a yellow color [111,112].

**Figure 4:**
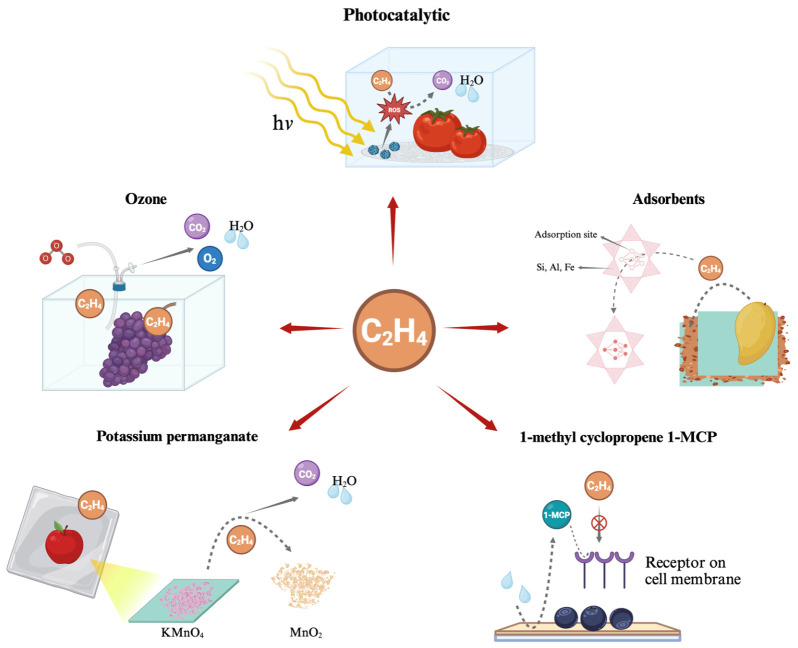
Schematic representation of the main ethylene (C_2_H_4_)-scavenging and -inhibiting systems applied in active food packaging. C_2_H_4_ present in the package headspace can be removed or neutralized through (i) photocatalytic degradation under light irradiation (hν), generating reactive oxygen species (ROS) that oxidize ethylene to CO_2_ and H_2_O; (ii) ozone treatment, where O_3_ reacts with C_2_H_4_ via ozonolysis; (iii) chemical oxidation using potassium permanganate (KMnO_4_), producing MnO_2_, CO_2_, and H_2_O; (iv) physical adsorption by porous materials such as clays and zeolites through surface adsorption and cation exchange; and (v) inhibition of ethylene action by 1-methylcyclopropene (1-MCP), which blocks C_2_H_4_ receptors on the cell membrane and delays ripening. Created in BioRender. Ruggeri, M. (2026). https://BioRender.com/f74bma4 (accessed on 27 May 2026).

Ethylene-scavenging systems can work with both chemical and physical methods by absorbing, adsorbing, and/or oxidizing the products. Ethylene scavengers are available as exogenous ethylene oxidants such as potassium permanganate (KMnO_4_) and ozone (O_3_), ethylene inhibitors (1-methyl cyclopropane, 1-MCP), ethylene adsorbents (zeolites and clays), and, more recently, emerging effective and environmentally friendly techniques based on catalytic oxidation of ethylene such as titanium oxide (TiO_2_) [50,113,114,115]. Other technologies available include activated alumina metal oxides and carbon with various metal catalysts, activated charcoal impregnated with palladium catalyst, and electron-deficient nitrogen-containing trienes [114,116]. A schematic overview of the ethylene removal and inhibition technologies discussed in this section is shown in Figure 4.

Traditionally, the most used ethylene scavenger, mainly in climacteric fruit, is potassium permanganate, which oxidizes ethylene to carbon dioxide and water, releasing manganese dioxide (MnO_2_) and potassium hydroxide (KOH).

The general stoichiometric oxidation reaction proceeds as in Equation (22) [107]:

(22) $${3C}_{2}H_{4}+12KMnO_{4}+2H_{2}O\overset{H_{2}O}{\to }12MnO_{2}+12KOH+H_{2}O+6CO_{2}$$

KMnO_4_ is often used in the form of ethylene (C_2_H_4_)-permeable sachets, blankets, or tubes incorporated into packages of fresh produce. However, maintaining its efficiency can be challenging, as scavenging performance may decrease over time due to saturation. In addition, safety concerns arise from the inherent toxicity and deep purple color of KMnO_4_, with the potential risk of accidental migration onto fresh products [115,117]. Although the quantities typically used in active packaging applications are generally low, reducing the likelihood of significant consumer exposure, KMnO_4_ remains a relevant safety concern due to its strong oxidizing nature. To minimize direct food contact, KMnO_4_ is commonly immobilized on porous inert supports (e.g., microporous mineral particles) and incorporated into sachets or separated active systems. Nevertheless, accidental migration or improper handling cannot be completely excluded, particularly if the active system is damaged or misused. Therefore, appropriate packaging design and safety assessment remain essential to ensure the safe application of KMnO_4_-based food packaging systems [117,118,119,120]. For these reasons, novel formulation strategies are being explored, including nanocomposites and carrier materials capable of increasing the effective surface area of KMnO_4_, enhancing ethylene scavenging performance, and improving handling safety [121].

Wang et al. (2022) [118] developed ethylene-scavenging films by combining KMnO_4_ with pumice, a porous volcanic rock that physically adsorbs ethylene and serves as a support to increase the specific surface area of KMnO_4_ exposed to the gas. The KMnO_4_–pumice composite was dispersed within a low-density polyethylene (LDPE) matrix. The study demonstrated that low KMnO_4_ loadings (1 wt. % and 3 wt. %) enhanced the films’ mechanical properties, crystallinity, and gas barrier performance against oxygen and water vapor. Moreover, the film containing 3 wt. % KMnO_4_ effectively preserved avocados for up to 20 days at 25 °C, suppressed ethylene and carbon dioxide (CO_2_) accumulation in the package headspace, and reduced the loss of flesh firmness. Ebrahimi et al. (2021) [122] prepared and optimized nanocomposites based on a polyolefin elastomer (POE) incorporating impregnated nanoparticles––nano silica (NS) and nano clay (NC)––loaded with KMnO_4_ using response surface methodology (RSM). The ethylene absorption capacity increased with higher concentrations of impregnated nanoparticles, attributed to the greater KMnO_4_ content. Furthermore, the optimized nanocomposite films effectively extended the shelf life of bananas for up to 15 days under ambient conditions.

Ozone treatment is considered a non-thermal method of food preservation that improves food safety without compromising quality and endangering the environment [123]. Since ozone is a highly unstable molecule that auto-decomposes spontaneously and quickly into oxygen atoms at room temperature, it does not accumulate substantially without continual generation [124]. O_3_ acts as an ethylene scavenger by reacting rapidly with C_2_H_4_ through ozonolysis, thereby removing ethylene from air, as shown in Equations (23)–(25) [125]:

C_2_H_4_ + O_3_ → 2HCHO (primary ozonolysis reaction) (23)

Subsequent oxidation reactions in excess of ozone/air:

HCHO + O_3_ → HCOOH + O_2_ (oxidation of formaldehyde) (24)

HCOOH + O_3_ → CO_2_ + H_2_O + O_2_ (oxidation of formic acid) (25)

Overall reaction:

C_2_H_4_ + 3O_3_ → 2CO_2_ + 2H_2_O + 3O_2_

Ozone treatments can be applied in both gaseous and aqueous phases, depending on the type of produce to be treated and the method of application [126]. Kartika et al. (2025) [125] demonstrated the potential of ozone treatment as a postharvest solution for extending the shelf life and maintaining the quality of jackfruit. Indeed, ozone treatment at 325–350 ppm for 60 min significantly reduced fruit rot disease and delayed ripening and senescence both directly, through its antifungal effect on pathogens, and indirectly, by inducing plant defense enzymes, as well as increasing total phenolic content. Additionally, ozone treatment suppressed ethylene production by inhibiting ethylene biosynthesis, in addition to reducing the respiration rate. Aqueous ozone (1.4 mg·L^−1^) treatment could effectively reduce ethylene production and improved the overall quality of fresh-cut cabbage during storage at 4 °C for 12 days. The effect observed may be ascribed to the strong oxidation of ozone inactivating the related enzymes in ethylene synthesis, thereby blocking ethylene biosynthesis [126]. Gaseous ozone treatment (0.15 ppm gaseous O_3_ during the day and 0.3 ppm overnight) during storage at 6 °C for 13 days was effective in ethylene removal, decreasing ethylene biosynthesis and delaying fruit softening in cantaloupes by maintaining their quality for longer [127].

Although ozone is an effective oxidizing agent for ethylene degradation and has shown promising applications in postharvest preservation, long-term or excessive ozone treatments have been associated with toxic effects, oxidative damage, and deterioration of sensory and textural characteristics. Therefore, ozonation conditions should be carefully adjusted according to the specific characteristics of each food product, with strict optimization of treatment parameters, including ozone concentration, exposure time, and product sensitivity, to minimize oxidative deterioration. Furthermore, regulatory acceptance of ozone-based food treatments remains a widely debated issue, which may limit broader commercial implementation. Nevertheless, when applied under controlled conditions and potentially combined with complementary preservation strategies such as refrigeration or modified atmosphere packaging, ozone represents a promising approach for postharvest quality preservation [121,128,129,130].

1-MCP is the most widely used commercial ethylene-binding inhibitor, which, applied exogenously as a gas, is being used to enhance the shelf life of fresh produce [122]. Treatment of freshly harvested cabbages with 1 μL·L^−1^ 1-MCP for 12 h followed by storage for 8 d at 25 ± 1 °C significantly extended the shelf life by inhibiting ethylene production and respiration rate. In particular, postharvest deterioration was reduced, chlorophyll degradation was retarded, and lipid peroxidation and accumulation of nitrite were inhibited [131]. Li and collaborators (2022) [132] investigated the synergistic preservation effect of 1-MCP and laser microporous film (LMF) on honey peaches. Fumigation of peaches with 2 μL·L^−1^ 1-MCP for 24 h followed by packing in LMF extended their shelf life to 28 days at 5 °C. In particular, the co-application of 1-MCP and LMF significantly inhibited the respiration rate, reduced the degradation of cell wall materials, and delayed ROS accumulation and membrane lipid peroxidation in honey peaches during cold storage, eventually slowing the softening of the peaches.

Ethylene adsorbents can be successfully incorporated into the packaging system by providing a porous inert material with a high surface area such as clay or zeolite [107]. A novel mineral- and clay-based ethylene scavenger for preserving fresh fruits and vegetables was developed by mixing sillimanite (Al_2_SiO_5_) and bentonite (Al_2_H_2_O_6_Si). The addition of bentonite significantly improved the adsorption capacity of sillimanite due to high surface area and pore volume, which provided physical and chemical intersection of ethylene molecules by increasing the rate of cation exchange. The developed system proved to be efficient and exhibited higher ethylene adsorption than commercial ethylene scavengers, in addition to being environmentally friendly and safe [133]. Another sustainable active material packaging was developed based on silver-impregnated-zeolite incorporation into chitosan Kraft paper coating (NaY-AgChKr) to prolong the shelf life of cherry tomatoes. Zeolite (NaY) was modified by cation exchange with silver ion (NaY-Ag) and incorporated into a chitosan filmogenic matrix, forming an active coating on the Kraft paper surface. The efficiency of the ethylene scavenger was evaluated for 30 days at 25 ± 2 °C. The developed active packaging proved to have a prominent role in delaying the ripening of cherry tomatoes and controlling the ethylene concentration in the headspace (15 ppm for NaY-AgChKr vs. 100 ppm for the control at 15th day) [134].

Titanium dioxide (TiO_2_) is a useful photocatalyst because of is chemical stability, nontoxicity, cost-effectiveness, and safety and is considered to be an attractive ethylene scavenger because, once exposed to UV light, TiO_2_ induces ethylene photodegradation by generating strong ROS on its surface that further react with ethylene and/or polyunsaturated phospholipids in the cell membranes of microorganisms [135]. In this context, cellulose nanofiber-based ethylene-scavenging antimicrobial films incorporating TiO_2_ nanoparticles were developed to extend the shelf life of tomatoes. In this case, to provide titanium nanotubes with photocatalytic function in visible light, TiO_2_ nanoparticles were modified with Cu_2_O. The novel active packaging showed efficient photocatalytic activity under visible light, resulting in a strong bactericidal effect and ethylene-scavenging activity, delaying fruit ripening, and extending fruit shelf life [136]. Polyethylene foam nets coated with a gelatin–TiO_2_ photocatalytic nanocomposite were developed to degrade the ethylene produced by papayas. Fruits wrapped in this novel active packaging exhibited 60% lower ethylene accumulation than the control after 4 days under UV-A light, owing to the photocatalytic degradation of ethylene, as well as the reduced autocatalytic ethylene production by the papayas. In particular, a decrease in respiration rate was detected during the climacteric peak, while no scalding or superficial fungal growth was observed. Moreover, the treated fruits showed a more pronounced preservation of green color and firmness compared with the control samples [137].

A summary of the described ethylene scavenger technologies is presented in Table 4. The table reports active compounds and composites, packaging configurations, removal mechanisms, quantitative performance, storage parameters, and corresponding food applications.

While ethylene-scavenging systems are effective in extending the postharvest shelf life of fruits and vegetables and reducing food loss and waste, their environmental profile varies considerably depending on the active materials employed [138]. Conventional oxidizing systems based on KMnO_4_ present limitations related to toxicity, single-use disposal, and restricted direct food-contact applications. In response, increasing attention has been directed toward alternative approaches, including photocatalytic systems, porous adsorbents, and biodegradable active packaging materials. Recyclable adsorbents and bio-based polymer matrices may offer advantages in reducing environmental burden, although challenges related to cost, scalability, regeneration efficiency, and material safety still limit broader implementation [138,139,140].

#### 3.1.3. Carbon Dioxide Scavengers and Emitters

Carbon dioxide gas (CO_2_) is commonly added in food MAPs, in packages of various perishable foods such as fish, meat, bakery, and dairy products. Properly high concentrations of CO_2_ display antibacterial properties, while in packages of fresh produce, CO_2_ produced from the product is equilibrated at relatively low levels to keep the freshness by suppressing physiologically reactive deterioration [141,142]. Inserting CO_2_-generating systems into the package environment is a practice in MAP technology, as a higher CO_2_ concentration is helpful for sensory aspects and retards unfavorable reactions. Products that can benefit from high CO_2_ levels include meat, poultry, fish, cheese, and baked goods [143]. However, the accumulation of excess CO_2_ in the package––above 20% for most commodities––generally leads to detrimental effects to the quality of the product, causing injuries such as browning and off-odor development, or to the integrity of the package, due to problems of pressure build-up and volume expansion [50,141,144,145,146]. Therefore, regardless of the specific benefits, the package atmosphere should be carefully controlled to maintain the desired CO_2_ concentration, which must be balanced or harmonized with appropriate levels of oxygen and nitrogen [141].

**Figure 5:**
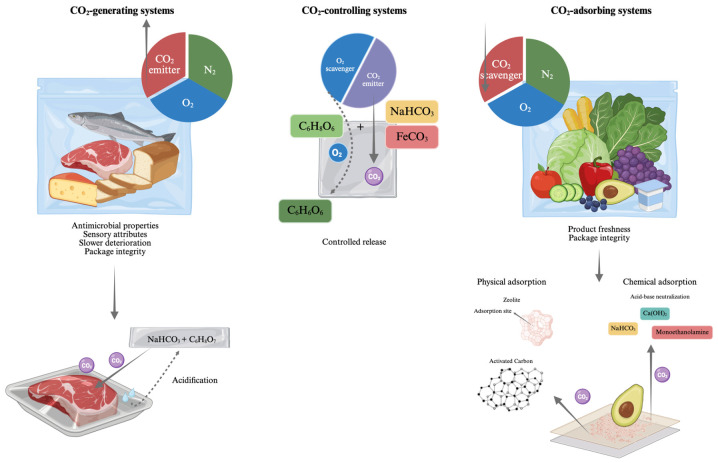
Overview of carbon dioxide (CO_2_) active packaging technologies for food preservation. The figure illustrates (i) CO_2_-generating systems, based on acid–base reactions (e.g., NaHCO_3_ with organic acids) that release CO_2_, helping to extend shelf life by inhibiting microbial growth, reducing drip loss, and preventing packaging shrinkage; (ii) CO_2_-controlling systems, combining CO_2_ emitters with oxygen scavengers to enable controlled release and headspace regulation; and (iii) CO_2_-adsorbing systems, which remove excess CO_2_ through physical adsorption (e.g., zeolites, activated carbon) or chemical absorption (e.g., alkaline compounds, salts, or oxides), thereby preserving product freshness and package integrity. Created in BioRender. Ruggeri, M. (2026) https://BioRender.com/rcslszp (accessed on 28 May 2026).

Maintaining high CO_2_ concentrations in food packaging through the use of CO_2_ emitters helps extend shelf life by suppressing microbial proliferation, displacing some or all of the O_2_ required for bacterial metabolism, and preventing shrinkage or collapse of semi-rigid plastic packages that are flushed with CO_2_-enriched nitrogen atmospheres [141]. Carbon dioxide emitters are often coupled to oxygen scavengers, since the oxygen adsorbed by the oxygen scavenger is directly replaced with carbon dioxide, balancing the levels of both gases in the package environment [141]. An overview of CO_2_-based active packaging technologies is presented in Figure 5.

The oldest formulation of a CO_2_ emitter is ferrous carbonate (FeCO_3_), which produces carbon dioxide by consuming oxygen in acidic environments. Since ferrous carbonate is highly unstable at ambient conditions, it is generally combined with metal catalysts and oxygen scavengers [141]. Sodium bicarbonate (NaHCO_3_) is commonly utilized as a CO_2_-releasing technology in combination with an organic acid, such as citric acid or ascorbic acid [147,148,149]. Sodium bicarbonate (NaHCO_3_) can react with citric acid under moisture, as shown in Equation (26) [141]:

3NaHCO_3_ + C_3_H_4_OH(COOH)_3_ → C_3_H_4_OH(COONa)_3_ + 3CO_2_ + 3H_2_O (26)

Active packaging using a CO_2_-emitter sachet was evaluated to investigate the shelf life of chicken fillets stored at 4 °C. The CO_2_-emitter sachet or pad, placed underneath the meat, is designed to absorb drips consisting of water and sarcoplasmic proteins. Upon absorption of the exudate, the sodium bicarbonate and citric acid contained in the emitter dissolve in the liquid. The citric acid creates an acidic environment, leading to the formation of undissociated carbonic acid, which shifts the equilibrium toward the production of water and CO_2_. The generated CO_2_ gas ensures that the CO_2_ concentration in the headspace remains high, thereby preventing package collapse. This effect counteracts compression of the meat and significantly reduces drip loss. The results demonstrated that packages containing a CO_2_ emitter did not collapse, showed no squeezed appearance, and exhibited limited drip loss. Furthermore, the CO_2_ emitter enhanced the shelf life of chicken fillets by inhibiting the growth of spoilage microorganisms (e.g., *Brochothrix* spp., *Pseudomonads* spp., *Enterobacteriaceae* spp., and lactic acid bacteria (LAB)) and by reducing the development of off odors over time [150].

Vacuum packaging or MAP combined with a CO_2_-emitter pad was developed and evaluated for its ability to prolong the shelf life of raw fish. The study demonstrated that the microbial and sensory quality of both vacuum-packaged and MAP raw fish was better preserved when a CO_2_ emitter was included. Furthermore, vacuum packaging combined with a CO_2_ emitter preserved quality as effectively as MAP. In particular, shelf life was extended by approximately 2 to 3 days when comparing “MAP + CO_2_ emitter” with “MAP alone” (approximately 13 days versus 9 days) and when comparing “Vacuum + CO_2_ emitter” with “Vacuum alone” (9 days versus 7 days) [151].

One of the latest works reported in the literature explored the feasibility of biodegradable films using poly(butylene adipate-co-terephthalate) (PBAT) and thermoplastic starch (TPS) for use as antimicrobial absorbent sachets and CO_2_ emitters in sustainable active packaging. The addition of zinc oxide (ZnO) nanoparticles (3 wt. % and 5 wt. %) to the packaging material was evaluated for its ability to impart antimicrobial properties, reduce the water sensitivity of PBAT-TPS active sachets, and hinder CO_2_ molecular transport through the film. The optimal combination ratio of citric acid (CA) and sodium bicarbonate (SB) (40:60) was determined to achieve the maximum CO_2_ generation within the sachet. All films exhibited a rapid initial CO_2_ release, followed by a slower release rate after 36 h, ultimately reaching equilibrium at 84 h. After equilibrium was reached, all samples exhibited a similar CO_2_ concentration in the headspace, at approximately 35%. The slow-release kinetics of CO_2_ gas from the sachet can help prevent shrinkage and excessive internal pressure while minimizing drip loss caused by the squeezing effect. Accordingly, the study demonstrated the potential of novel bio-nanocomposite films to regulate CO_2_ release behavior in accordance with the needs of packaged food [152].

In CO_2_-emitting packaging systems, ascorbic acid combined with sodium bicarbonate serves a dual function: it acts as an acidifying agent that promotes CO_2_ generation from sodium bicarbonate and as an oxygen scavenger by oxidizing to dehydroascorbic acid, consuming oxygen at a stoichiometric ratio of 1 mol O_2_ per 2 mol of ascorbic acid [56,149]. Technologies that combine sodium bicarbonate and ascorbic acid are commercially available; examples include the Verifrais™ CO_2_-emitting package (SARL Codimer, Paris, France), multi-function AGELESS^®^ GE (Mitsubishi Gas Chemical, Tokyo, Japan), and FreshPax^®^ (Multisorb Technologies, Buffalo, NY, USA), which are implemented as combined O_2_-scavenger/CO_2_-emitter systems for extending the shelf life of perishable foods [1]. Also, ascorbic acid coupled with ferrous carbonate or iron carbonate can be used to release carbon dioxide or sodium ascorbate (C_6_H_7_O_6_Na), which react with organic acid under wet conditions to produce CO_2_ [141].

Fermented dairy products such as yogurt, fermented vegetables, roasted coffees, yams, and miso produce significant amounts of CO_2_, which has detrimental effects [141,146]. Unless removed, the progressive increase of the internal pressure of CO_2_ is responsible for ballooning or even bursting of the package, which finally collapses [153,154,155]. Thus, CO_2_ absorbers can remove the excess CO_2_ and control the CO_2_ concentration in food packages [146]. CO_2_ adsorption or removal based on chemical reactions depends on the interaction between alkaline solutions, salts, or oxides and CO_2_ through acid–base neutralization mechanisms [154]. In contrast, physical CO_2_ adsorbents capture CO_2_ according to an equilibrium relationship, in which the amount of adsorbed CO_2_ increases with its partial pressure in the package headspace [154]. CO_2_ absorbers include chemical adsorbents such as monoethanolamine, calcium hydroxide, calcium oxide, and sodium carbonate, as well as physical adsorbents such as activated carbon and zeolites [156,157]. Chemical adsorbents generally exhibit excellent adsorption capacity but often have slower reaction rates, require moisture activation, and involve largely irreversible reactions [141]. Physical adsorbents, on the other hand, offer rapid and reversible adsorption governed by adsorption isotherms and influenced by moisture; however, they typically exhibit weaker adsorption capacity compared to chemical adsorbents [141,157].

Calcium hydroxide is the most commonly used chemical adsorbent and performs a thermodynamically spontaneous reaction, producing safe reaction products, as shown in Equation (27) [141]:

Ca(OH)_2_ + CO_2_ → CaCO_3_ + H_2_O (27)

Activated carbon, with respect to zeolite, adsorbs lesser amount of carbon dioxide. Zeolite’s potential to adsorb CO_2_ is greatly disturbed or decreased by moisture, in contrast to activated carbon, for which CO_2_ adsorption is relatively constant regardless of its moisture adsorption [140]. Chemical adsorbers such as calcium hydroxide an excellent performance but a slow irreversible reaction rate, while physical adsorbents show a quick and reversible reaction rate but have weak adsorption power [141].

A summary of the described carbon dioxide active technologies is presented in Table 5. The table includes active materials, carrier formats, mechanisms of action, performance outcomes, storage conditions, and tested food commodities.

Given their effectiveness in maintaining the desired package atmosphere for perishable foods, increasing attention has been directed toward enhancing the sustainability of CO_2_-emitter and -scavenger systems, particularly through the development of biodegradable and bio-based active packaging materials [152,159]. In parallel, porous CO_2_ adsorbents derived from biomass or waste materials have emerged as promising alternatives, due to their renewable origin and potential for regeneration and reuse, supporting the integration of active packaging functionality with more sustainable material design [159,160].

### 3.2. Moisture-Absorbing and -Controlling Systems

Food moisture content and water activity (a_w_) are two fundamental parameters impacting food chemical stability [161]. Indeed, the presence of water makes many ingredients susceptible to chemical reactions and microbial growth and can also influence the physical characteristics of solid food. For example, aspartame, a widely used sweetener, is incorporated into a broad range of high- and low-moisture food products. The effect of moisture on aspartame stability has been shown to correlate with both water activity—where higher water activity levels accelerate the degradation rate of aspartame—and the temperature sensitivity of the reaction [161,162]. Moisture content can significantly affect vitamin stability, as the degradation of ascorbic acid and thiamine, as well as the rate of riboflavin degradation, increases with rising water activity, thereby having detrimental effects on the nutritional profile of the original product [161,163]. Moisture can also act as a substrate for lipid oxidation and participate in the formation of volatile compounds [164,165,166]. The water activity is also responsible for both enzymatic and non-enzymatic browning, leading to loss of nutrients, caramelization, ascorbic acid degradation, and formation of brown pigments [167]. In low-moisture particulate products, residual moisture not only affects both chemical and physical stability, but also contributes to phase transformations, reduced powder flowability, or powder caking and clumping [168]. Moisture content, like water activity, has a significant effect on microbial growth. High-moisture foods (a_w_ > 0.85) are highly perishable foods, as they are susceptible to the growth of spoilage and pathogenic microorganisms. Microbial spoilage of intermediate-moisture foods is a relatively slow process and is mainly caused by yeasts and molds. Low-moisture foods are generally not subjected to microbial spoilage unless their moisture content increases. Microbial growth ceases at water activity values below the minimal value (a_w_ < 0.60), but the microbes do not die immediately and may remain dormant in the food for prolonged periods [27].

Food susceptible to moisture must be packaged with a moisture-controlling sachet or pad to prevent moisture absorption by the product or condensation responsible for microbial spoilage and/or low consumer appeal [50]. In fact, the storage of fresh food products in moist and warm environments is the cause of mold spoilage. The accumulation of excessive relative humidity due to moisture in packages can cause caking of powdered products, softening of dry crispy products, or moistening of hygroscopic products. On the contrary, excessive moisture loss from food products through the packaging material can result in product desiccation or favor lipid oxidation. Moisture-control agents and films with the appropriate water vapor permeability are employed to lower the water activity and to establish the desired relative humidity in the package headspace [50,162,169].

Moisture absorbers comprise various hygroscopic substrates or substances that can be broadly classified in relative humidity (RH) controllers and moisture removers. RH controllers absorb humidity from the package headspace and include materials such as desiccants, whereas moisture removers are designed to absorb liquid exudates released directly from food products [169]. Traditionally, moisture absorbers have been available as inorganic-based systems, commonly in the form of sachets containing calcium oxide (CaO), silica gel, calcium chloride (CaCl_2_), potassium chloride (KCl), potassium carbonate (K_2_CO_3_), and natural clays such as zeolite, montmorillonite, and bentonite [169,170,171]. More recently, research attention has shifted toward the development of novel superabsorbent composite materials, including both organic- and polymer-based systems [169]. Organic-based moisture absorbents mainly include cellulose and its derivatives, such as carboxymethylcellulose, as well as fructose and sugar alcohols such as sorbitol and xylitol [169,172]. Polymer-based moisture absorbers that have been extensively investigated include starch copolymers, chitosan biopolymers, polyvinyl alcohol, and other synthetic or semi-synthetic resins [173,174,175].

Among the moisture-controlling agents developed so far, desiccants such as calcium chloride (CaCl_2_) exhibit a particularly high moisture-holding capacity. However, a major drawback of CaCl_2_ is its tendency to liquefy upon moisture absorption [176]. To overcome this limitation, Boonsiriwit et al. (2022) [177] demonstrated the feasibility of producing a solid hygroscopic material by combining CaCl_2_ with an acid-modified natural clay, such as vermiculite. The resulting hygroscopic agent (AEV/CS40) was shown to effectively preserve the quality of white button mushrooms by delaying deterioration in terms of color, firmness, pH, and microbial growth. Silica gel, in contrast, remains in a crystallized form during moisture adsorption, although it exhibits a lower desiccation rate, and is thus useful only in dry food systems. Other desiccants for dried food applications include molecular sieves, calcium oxide (CaO), and natural clays such as montmorillonite [178,179].

For foods with high water activity such as fish, meat, poultry, fruits, and vegetables, the control of excess moisture requires the use of moisture removers rather than traditional desiccants. Moisture removers typically interact with moisture through the physical adsorption of liquid or vapor-phase water molecules from the surrounding environment. One increasingly popular approach for moisture regulation is the use of superabsorbent polymers. Examples include rice starch-grafted sodium acrylate super absorbent material [180], wood fiber-based absorbent pads containing polyvinyl alcohol (PVA) [181], and superabsorbent aerogel developed through a Schiff base reaction between gelatin and dialdehyde starch and reinforced with bacterial cellulose [182].

Despite their advantages, starch-based materials present several limitations, including poor mechanical properties, low water stability, high moisture sensitivity, and weak moisture barrier performance. Therefore, plasticizers and nanofillers are commonly incorporated into thermoplastic starch-based films to improve hydrophobicity and enhance mechanical strength [183,184]. Reported strategies include the incorporation of microcrystalline cellulose into starch-based films [185], the production of starch–chitosan composite films [186], and the development of nano clay–starch nanocomposites [187,188]. In addition to films, water-absorbable and reusable starch-based aerogels produced from different starch sources such as potato, cassava, bean, and maize have demonstrated a high capacity to remove exudates and liquids released by chicken meat, thereby slowing down the spoilage process [189]. Another moisture-control strategy involves intercepting water in the vapor phase by placing humectants such as fructose between two layers of plastic film with high water vapor permeability, or by using moisture-absorbent sachets [169,190,191].

Given the essential role of moisture regulation in maintaining food quality, increasing attention has been directed toward developing more sustainable moisture-control systems compatible with biodegradable and bio-based packaging. The transition toward compostable and renewable alternatives has, however, introduced new challenges due to the generally higher moisture permeability of many biopolymers [192,193]. This has stimulated the development of integrated moisture-control strategies, including bio-based absorbents and compostable desiccant systems, aimed at combining preservation performance with reduced environmental impact. In this context, natural desiccants derived from renewable or waste-based resources have emerged as promising alternatives to conventional synthetic moisture absorbers due to their biodegradability, lower environmental burden, and potential valorization of agro-industrial by-products. Such approaches may support the broader adoption of sustainable packaging by improving the functionality of biodegradable systems in moisture-sensitive food applications [193,194,195,196].

### 3.3. Antimicrobial Packaging

Antimicrobial packaging is a form of active packaging designed to extend food shelf life by reducing or inhibiting the growth of microorganisms present in the food or packaging environment. Antimicrobial agents may be gradually released from the packaging matrix into the surrounding environment or act through non-migratory mechanisms at the food–package interface [197]. Their antimicrobial activity depends on the target microorganism and may involve disruption of membrane integrity, inhibition of essential metabolic pathways, interference with nucleic acid synthesis, or impairment of ribosomal function [198,199]. Various antimicrobial strategies have been developed, including controlled-release, adsorption-based, and immobilized systems [200,201]. Among commercially available systems, sachets or pads containing volatile antimicrobial agents such as chlorine dioxide, ethanol, and sulfur dioxide remain among the most widely adopted solutions [202].

Natural antimicrobial compounds have attracted increasing attention as alternatives to synthetic preservatives due to their broad-spectrum antimicrobial activity and compatibility with sustainable packaging systems. Plant-derived antimicrobials, including essential oils from cinnamon, allspice, clove, thyme, rosemary, and oregano, as well as extracts from onion, garlic, radish, mustard, and horseradish, contain bioactive compounds such as phenolics, terpenoids, organosulfur compounds, and isothiocyanates [198,200,203]. Their antimicrobial effects are mainly associated with disruption of microbial membranes, increased permeability, leakage of intracellular constituents, enzyme inhibition, interference with nucleic acid synthesis, and oxidative stress induction, ultimately leading to microbial growth inhibition or cell death [187,190]. However, their direct application in food systems may be limited by hydrophobicity, volatility, chemical instability, and potential adverse sensory effects at effective concentrations. To overcome these limitations, several incorporation strategies have been developed, including encapsulation, emulsification, nano- and micro-carrier systems, edible coatings, and active packaging matrices [201,204].

Recent studies have demonstrated the effectiveness of these approaches. Chen et al. (2024) [205] developed a silk fibroin/chitosan edible antimicrobial coating incorporating a 3% (v/v) cinnamon essential oil microemulsion, which exhibited inhibition rates exceeding 98% against bacterial and fungal spoilage microorganisms and extended strawberry shelf life from 2 to 8 days. Similarly, Kraśniewska and Gniewosz (2025) [206] developed a polyethylene terephthalate/polypropylene (PET/PP) film coated with pullulan-containing clove essential oil, which exhibited sustained antibacterial activity against *Escherichia coli*, *Salmonella enteritidis*, *Staphylococcus aureus*, and *Listeria monocytogenes* when applied to spinach stored at 4 °C, without negatively affecting visual quality. Fan et al. (2024) [207] also reported that oregano essential oil-loaded zein–pectin–chitosan nanoparticles improved antimicrobial stability and extended the shelf life of red sausage up to 9 days.

In addition to plant-derived compounds, other natural antimicrobial agents have also been investigated for food preservation and antimicrobial active packaging applications. Animal-derived antimicrobials include lysozyme, lactoperoxidase, lactoferrin, and antimicrobial peptides, while algae-derived compounds include alginate, carrageenan, fucoidan, phlorotannins, and other bioactive metabolites with antimicrobial potential [203,208,209,210,211]. Microbial-derived antimicrobials include natamycin, organic acids, diacetyl, hydrogen peroxide, and bacteriocins such as nisin, pediocin, and reuterin, which are particularly effective against spoilage and pathogenic microorganisms [201,212]. Among these, nisin remains the only commercially approved bacteriocin widely used in food systems, whereas natamycin is primarily employed for controlling yeasts and molds [200]. In addition, naturally occurring antimicrobial fatty acids such as lauric acid have also been explored for food preservation applications [213].

Among natural antimicrobial biopolymers, chitosan has attracted considerable interest due to its biodegradability, non-toxicity, excellent film-forming properties, and intrinsic antimicrobial activity against bacteria, fungi, and algae [214]. Gao et al. (2022) [215] developed a chitosan-based edible film incorporating ε-polylysine hydrochloride and nisin that improved mechanical and barrier properties, exhibited over 90% inhibition against *Staphylococcus aureus* and *Escherichia coli*, and prolonged the shelf life of fresh peaches. More recently, Xu et al. (2025) [216] developed a rechargeable antimicrobial packaging film based on ε-poly-L-lysine stabilized with phase-transitioned lysozyme, achieving complete inactivation of *S. aureus* and *E. coli* within 30 min and maintaining efficacy after 10 recharge cycles.

Additional antimicrobial strategies include organic acids such as sorbate, propionate, and benzoate, whose antimicrobial activity is associated with intracellular acidification and disruption of microbial metabolic functions [201,217]. Chelating agents such as EDTA can destabilize the lipopolysaccharide layer of Gram-negative bacteria, increasing membrane permeability [202]. Inorganic antimicrobial agents, including silver-substituted zeolites, have also been incorporated into active packaging systems intended for direct food contact [218,219]. Furthermore, oxygen-scavenging systems can reduce oxygen concentrations within the package, thereby limiting the growth of aerobic microorganisms and molds [220].

Despite their promising potential, antimicrobial active packaging systems still face several challenges, including the selection of effective antimicrobial concentrations, controlled release from packaging matrices, preservation of antimicrobial activity during storage, compatibility with different food matrices, and the potential development of microbial resistance following prolonged exposure [221]. Some of these limitations may be partially overcome by exploiting synergistic interactions between antimicrobial compounds, which can enhance antimicrobial efficacy, broaden the spectrum of activity, and potentially limit the emergence of resistant microorganisms.

A summary of the described antimicrobial active packaging technologies is presented in Table 6. The table includes active materials, carrier formats, performance outcomes, storage conditions, and tested food commodities.

The development of antimicrobial active packaging is increasingly moving beyond preservation performance alone toward the integration of more sustainable material solutions [225]. Biodegradable and bio-based polymer matrices such as starch, cellulose, chitosan, alginate, polylactic acid (PLA), and polyhydroxyalkanoates (PHAs), together with antimicrobial compounds derived from plant extracts, essential oils, peptides, or agro-food by-products, represent promising alternatives to conventional petroleum-based packaging. In addition, greener extraction methods and biodegradable carrier systems are being explored to reduce the environmental footprint associated with the production and incorporation of active agents, supporting the transition toward more circular food packaging solutions [226,227,228].

### 3.4. Antioxidant Packaging

Antioxidant active packaging is an active preservation strategy designed to delay oxidative deterioration in food systems by incorporating compounds capable of inhibiting or slowing oxidation reactions. Oxidative spoilage represents one of the major causes of food quality deterioration, particularly in lipid-rich and oxygen-sensitive foods, leading to rancidity, discoloration, nutrient degradation, and sensory deterioration. Antioxidant packaging films (APFs) have emerged as effective systems to mitigate oxidative damage by enabling controlled delivery of antioxidant compounds and improving preservation performance during storage [229,230]. Antioxidant agents can act as primary antioxidants, also referred to as chain-breaking antioxidants, by scavenging free radicals, or as secondary antioxidants, which prevent oxidation by deactivating hydroperoxides, chelating pro-oxidant metals, quenching singlet oxygen, or limiting oxygen availability [231,232,233].

Primary antioxidants include synthetic compounds such as butylated hydroxyanisole (BHA), butylated hydroxytoluene (BHT), and propyl gallate, as well as natural antioxidants including plant extracts, essential oils, phenolic compounds, flavonoids, tocopherols, and carotenoids. Due to increasing safety concerns associated with synthetic additives, naturally derived antioxidants have attracted considerable attention as safer and more sustainable alternatives [234]. Secondary antioxidants include chelating agents such as EDTA, citric acid, and lactoferrin; UV absorbers such as benzophenones, benzotriazoles, pigments, and phenolic-rich extracts; singlet oxygen quenchers including carotenoids, tocopherols, and polyphenols; and oxygen scavengers, which reduce oxygen availability within the package environment [234,235].

Different technological strategies have been developed for antioxidant active packaging systems. One common approach involves the use of independent active devices such as sachets, pads, or labels containing antioxidant agents, particularly oxygen scavengers based on iron or ferrous oxide powders. Alternatively, antioxidant compounds can be incorporated directly into polymeric matrices through casting, extrusion, or surface-coating processes, allowing controlled migration of active compounds toward the food product [229,234]. More recent developments have focused on micro- and nanoencapsulation technologies to improve antioxidant stability, compatibility with packaging matrices, and controlled release behavior, thereby enhancing long-term preservation performance [232,234]. Antioxidants may also be covalently immobilized onto polymer surfaces, exerting protective activity through direct contact without migration. In some systems, antioxidant activity can occur in the package headspace through radical scavenging mechanisms, reducing oxidative degradation even without direct contact with the food [235].

Recent studies have demonstrated the effectiveness of antioxidant active packaging systems. Barzan et al. (2024) [236] developed cellulose-based antioxidant active packaging incorporating natural extracts derived from grape marc, olive pomace, and *Moringa oleifera* leaves. The developed systems exhibited significant antioxidant activity and prevented more than 50% of lipid peroxidation in ground beef over 16 days of refrigerated storage, highlighting the potential of agro-industrial by-products as sustainable antioxidant sources. Similarly, Dong et al. (2022) [237] developed an antioxidant active packaging system based on nanoencapsulated natural antioxidants incorporated into polymeric matrices, demonstrating improved antioxidant stability, controlled release, and enhanced preservation performance. Freitas et al. (2022) [238] also reported that biodegradable antioxidant films containing rice straw extract effectively reduced oxidative spoilage and improved food preservation, further supporting the development of sustainable antioxidant packaging alternatives.

The effectiveness of antioxidant active packaging depends on several formulation and design parameters, including the optimal antioxidant concentration, compatibility between antioxidant compounds and polymeric substrates, stability during processing and storage, release kinetics, and interactions with the food matrix and packaging headspace [230,233,234,235]. Despite significant advances, challenges remain regarding the scalability of production technologies, preservation of antioxidant functionality under real storage conditions, and optimization of controlled-release performance [231,234].

A summary of the described antioxidant active packaging technologies is presented in Table 7. The table includes active materials, carrier formats, performance outcomes, storage conditions, and tested food commodities.

Antioxidant active packaging has also increasingly evolved toward more sustainable formulations through the replacement of synthetic additives with bio-based alternatives [243]. Natural antioxidants derived from plant extracts, lignin, phenolic compounds, and agro-food by-products such as winery residues, fruit pomace, or other lignocellulosic biomass represent particularly promising options, as they combine antioxidant functionality with renewable sourcing and waste valorization [243,244]. Their incorporation into biodegradable polymer matrices such as PLA, PHA, polybutylene succinate (PBS), polybutylene succinate adipate (PBSA), PBAT (polybutylene adipate terephthalate), or starch-based blends may support the development of more circular packaging systems, although the actual environmental benefits still require careful life cycle and end-of-life assessment [234,245].

### 3.5. Flavor/Odor Control

The mass transfer of components between food and packaging causes the loss of volatile flavors and aromas from food. The possible interactions that can happen among foodstuffs, polymer films, and the environment include permeation of oxygen, water vapor, carbon dioxide, and other gases; migration of monomers or additives; and adsorption (scalping) of aroma compounds, fats, organic acids, or pigments [246,247,248]. Migration is the transfer of substances from the package into the food due to direct contact. This event occurs mostly in plastic packaging systems, in which plastic additives are not chemically bound to the polymer molecules and thus move freely within the polymer matrix. Flavor scalping consists of the sorption of food aroma compounds by packaging materials. These events can negatively affect the flavor of food, reduce the product quality, or induce package system deterioration. In a positive way, flavor scalping can be useful to selectively adsorb unwanted odors and flavors [247,249]. The first aspect that should be considered to reduce scalping is the polymer selection. An approach contemplates the replacement of monomeric with polymeric plasticizer compounds of higher molecular weight with reduced tendency for migration. Flavors can be encapsulated to protect them from undesirable interactions, incorporated in packaging material to overcome flavor scalping, or selectively emitted to prevent off-flavor development. On the contrary, flavor and odor adsorbers scavenge unwanted gaseous molecules such as volatile package compounds, oxidative and biochemical metabolites of foods, microbial metabolite respiration products, or off-flavor contamination [250]. Examples of flavor and odor adsorbers include cellulose triacetate or acetylated papers (limonin absorbers), citric acid, activated carbon/zeolites/clays (aldehydes adsorbers), and ferrous/salt/ascorbate (volatile amine adsorbers) [60,251,252]. Commercial examples include SIAD’s Aroma+^®^ modified-atmosphere packaging, which combines food-grade gases with natural flavorings to preserve aroma and reduce undesirable volatiles, and ScentSational’s EncapScent^®^ microencapsulated aroma coatings that release scent upon handling; flavor enhancement solutions such as Corbion’s PuraQ^®^ Arome are also used commercially to improve sensory perception in reduced-sodium foods. The commercial use of flavor/odor adsorbers and releasers remains controversial due to their ability to mask natural spoilage reactions or develop the desired flavors and odors during storage. These agents can also aromatize the packaging material, attracting the potential costumer [252]. Table 8 presents an overview of the principal aroma and flavor systems, with emphasis on their function, mechanism of action, commercial implementation, and food applications.

A particularly attractive aspect of flavor and aroma active packaging lies in the possibility of coupling controlled release functionality with the use of bio-based carrier materials. Biopolymers such as starch, chitosan, pectin, alginate, cellulose derivatives, soy protein, gluten, and whey protein have been extensively investigated as matrices for essential oils and volatile bioactive compounds, offering tunable retention and release properties [138,255]. In addition to reducing dependence on conventional petroleum-based plastics, these systems create opportunities for the valorization of renewable resources and agro-industrial by-products, aligning active packaging design with more circular material strategies [248,255]. Emerging biotechnological approaches, including precision fermentation, may further support this transition by enabling the sustainable production of natural flavor compounds and bioactive ingredients from abundant substrates using engineered food-grade microorganisms, potentially reducing reliance on conventional extraction or synthetic production routes [256].

### 3.6. Multifunctional Systems

The evolution of active food packaging is increasingly moving toward multifunctional systems capable of simultaneously addressing multiple spoilage pathways within a single material platform. This shift reflects not only the growing complexity of food preservation requirements, but also the broader transition toward sustainable, bio-based packaging solutions [257,258]. A major catalyst for this development has been the increasing use of naturally derived active compounds. Unlike conventional synthetic additives, many bio-based materials inherently possess multiple biological functionalities. Plant extracts, essential oils, polyphenols, proteins, polysaccharides, and biomass-derived compounds frequently exhibit overlapping antioxidant, antimicrobial, antifungal, enzyme-inhibitory, metal-chelating, and film-forming properties [230,258,259]. Consequently, the search for safer, biodegradable, and clean-label preservation technologies has naturally accelerated the emergence of multifunctional active packaging systems, where a single active component or material matrix can perform several preservation roles simultaneously. This transition is also supported by the recognition that food spoilage is fundamentally a multifactorial process. Single-function active packaging strategies—such as oxygen scavengers, antimicrobial coatings, or ethylene absorbers—can effectively delay specific deterioration mechanisms but often fail to provide comprehensive protection under real storage conditions. Fresh fruits and vegetables, for instance, remain metabolically active after harvest and undergo concurrent respiration, senescence, moisture accumulation, microbial colonization, oxidative degradation, and enzymatic browning. Similarly, animal-derived foods are particularly susceptible to lipid oxidation, protein degradation, microbial spoilage, and water migration. These overlapping deterioration pathways necessitate integrated preservation approaches capable of simultaneously regulating multiple quality loss mechanisms [260,261,262].

Recent research increasingly reflects this systems-level approach. Rather than combining independent preservation technologies externally, multifunctional packaging architectures now integrate several active functions directly within a single material design. For example, Jilani et al. (2025) [262] developed a dual-function active packaging system combining antimicrobial release and CO_2_ scavenging for cherry tomato preservation, using β-cyclodextrin inclusion complexes loaded with linalool or eugenyl acetate together with polyethyleneimine as a CO_2_ scavenger. This integrated system significantly delayed fungal spoilage and improved firmness retention, demonstrating how coordinated regulation of microbial growth and package atmosphere can outperform conventional single-function approaches.

Biopolymer-based multifunctional systems represent another important direction. Agar-derived films incorporating sodium carbonate, sodium glycinate, and carvacrol have demonstrated simultaneous moisture absorption, CO_2_ scavenging, and antimicrobial activity, highlighting the feasibility of multifunctional inserts for MAP applications [261]. Similar strategies have emerged using biodegradable polysaccharide and protein matrices, where structural packaging functions are coupled with active preservation performance [263].

Nanotechnology has further expanded multifunctionality beyond what conventional natural extracts can achieve. Carbon dots (CDs), for example, combine antioxidant, antimicrobial, enzyme-regulating, and ROS-modulating activities within a single nanomaterial platform [257]. CD-based nanocomposite films have shown efficacy in preserving salmon, bananas, asparagus, and high-fat meat products through simultaneous inhibition of lipid oxidation, suppression of microbial growth, and reduction of enzymatic browning [264]. Likewise, nanozyme-based systems offer engineered multifunctionality by mimicking natural enzymatic activities while incorporating antimicrobial and stimuli-responsive behavior [265]. Table 9 provides a comparative overview of representative multifunctional active packaging systems, with emphasis on their functional roles, mechanisms of action, food applications, and degree of commercial implementation.

Despite their promise, multifunctional systems also raise important sustainability considerations. Greater functional complexity often requires composite architectures, encapsulation technologies, nanomaterials, or hybrid chemistries that may compromise recyclability, biodegradability, or regulatory acceptance. The use of metallic nanoparticles, synthetic carrier polymers, or multilayer structures may improve preservation efficacy while increasing environmental burden or complicating end-of-life management [230]. Therefore, the future of multifunctional active packaging should not be defined solely by performance enhancement, but by the ability to reconcile preservation efficiency with material circularity, safety, and scalable sustainable manufacturing. In this context, the most promising next-generation systems are likely to be those that combine multifunctionality with green material design—leveraging naturally multifunctional bioactive compounds, biodegradable carrier matrices, and low-impact fabrication strategies to deliver integrated, effective, and environmentally responsible food preservation solutions [258,274].

## 4. Final Remarks

Active packaging technologies have emerged as powerful tools to mitigate food loss and waste by actively addressing the major drivers of quality deterioration, including oxidation, microbial proliferation, moisture imbalance, uncontrolled gas exchange, and sensory degradation. As highlighted throughout this review, a broad range of strategies—including oxygen and ethylene scavengers, carbon dioxide regulators, moisture-control systems, antimicrobial and antioxidant packaging, and flavor/aroma management technologies—have demonstrated substantial potential to extend shelf life and improve food preservation. However, as the field continues to evolve, future efforts should focus on refining these systems to better address evolving sustainability, regulatory, and practical integration of active packaging technologies across diverse food systems.

First, the transition from conventional petroleum-based systems toward biodegradable and bio-based active packaging must be accelerated without compromising functional performance. Although biopolymers offer clear environmental advantages, material-specific challenges related to mechanical performance, moisture sensitivity, and barrier properties may still constrain their broader implementation in certain food packaging applications. Future research should focus on designing multifunctional biodegradable systems capable of matching the performance of conventional materials under realistic storage and distribution conditions.

Greater emphasis must be placed on the development of sustainable active agents and carriers. While conventional technologies often rely on metal-based scavengers, synthetic additives, or non-recyclable multilayer structures, emerging alternatives based on natural antioxidants, essential oils, enzymes, microbial systems, waste-derived adsorbents, natural desiccants, and precision fermentation-derived bioactives offer promising pathways toward greener active packaging. However, their scalability, reproducibility, and long-term stability require further validation.

Controlled release and system predictability remain major scientific challenges in active packaging. The performance of these systems is highly dependent on food composition, humidity, temperature, package geometry, and the interactions between active compounds and polymer matrices. Future studies should prioritize predictive modeling of release kinetics, migration behavior, adsorption/desorption equilibria, and active agent depletion under dynamic real-world storage and distribution conditions.

Safety and regulatory harmonization will remain critical, particularly for advanced active packaging materials. Nanostructured systems, encapsulated bioactives, and photocatalytic materials continue to raise questions regarding migration, toxicological safety, and regulatory approval. Standardized testing frameworks will be essential for broader commercial translation.

Industrial scalability and economic feasibility must be addressed more systematically. Many promising technologies remain confined to laboratory-scale demonstrations, while challenges related to manufacturing integration, processing compatibility, cost competitiveness, and supply chain implementation limit adoption. Closer collaboration between academia, packaging manufacturers, food producers, and regulatory stakeholders will be necessary to bridge this translational gap.

Finally, the ongoing convergence of active packaging with intelligent sensing, responsive release mechanisms, digital traceability, and circular material design is driving the development of increasingly multifunctional systems that combine preservation with monitoring and adaptive functionality.

Overall, the future of active packaging will depend not simply on replacing conventional materials, but on developing scientifically robust, economically viable, and environmentally responsible preservation systems that can be realistically implemented across modern food supply chains.

## Figures and Tables

**Table 1:** Comparative overview of major active packaging systems.

Active Packaging System	Mechanism of Action	Active Agents/Materials	Commodities	Main Limitations
O_2_ scavenging systems	Remove residual O_2_ from the package headspace or limit O_2_ ingress to reduce oxidative deterioration and aerobic microbial growth	Metal-based scavengers, ascorbic acid, enzymes (e.g., glucose oxidase/catalase), UV-activated scavengers, unsaturated hydrocarbons, redox systems, phenolic compounds, microorganisms	Meat, fish, bakery products, cheese, coffee, nuts, ready-to-eat foods	Limited efficiency depending on environmental conditions (e.g., humidity); possible migration of active compounds; activation requirements; cost
C_2_H_4_ scavenging systems	Remove C_2_H_4_ to delay ripening and senescence in climacteric produce	KMnO_4_, clays, zeolites, TiO_2_ photocatalysts, ozone-based systems	Fruits and vegetables	Toxicity concerns (e.g., KMnO_4_); regulatory limitations; oxidative damage (e.g., ozone exposure)
CO_2_ emitter/scavenger systems	Release or absorb CO_2_ to regulate respiration, microbial growth, and package atmosphere composition	Sodium bicarbonate/citric acid systems, calcium hydroxide, activated carbon, zeolite	Fresh meat, poultry, seafood, cheese, fresh produce	Excess CO_2_ may alter sensory quality; moisture dependence; limited control over release/absorption kinetics
Moisture control systems	Regulate moisture by absorbing excess water or controlling relative humidity to reduce microbial growth and preserve texture	Calcium oxide, calcium chloride, potassium carbonate, natural clays, silica gel, superabsorbent polymers, humectants	Fresh meat, fish, poultry, bakery products, fresh produce	Saturation over time; reduced effectiveness in high-moisture systems; disposal concerns
Antimicrobial packaging systems	Inhibit or reduce microbial growth through controlled release or contact-active mechanisms	Essential oils, organic acids, bacteriocins, silver nanoparticles, chitosan, enzymes	Meat, dairy, seafood, bakery products, fresh produce	Regulatory constraints; sensory alterations; migration concerns; stability of active compounds
Antioxidant packaging systems	Delay oxidative reactions through the release or immobilization of antioxidant compounds	Tocopherols, plant extracts, essential oils, butylated hydroxyanisole, butylated hydroxytoluene, polyphenols, metal chelators	Lipid-rich foods, meat, nuts, edible oils, snack products	Migration control; compatibility with polymeric substrates; release characteristics in real food systems
Flavor/odor control systems	Remove undesirable volatile compounds or release desirable aroma compounds	Activated carbon, citric acid, cyclodextrins, clays, zeolites, aroma releasers	Coffee, seafood, ready meals, fresh produce	Limited adsorption capacity; potential impact on desirable aroma profiles
Multifunctional systems	Combine multiple preservation functions within a single packaging system	Hybrid materials integrating scavenging, antimicrobial, antioxidant, and moisture-control functions	Ready-to-eat foods, fresh produce, meat, high-value food products	Increased complexity; higher cost; regulatory approval challenges; compatibility issues among active agents

O_2_ oxygen; C_2_H_4_ ethylene; KMnO_4_ potassium permanganate; TiO_2_ titanium dioxide; CO_2_ carbon dioxide.

**Table 2:** Classification categories of oxygen-scavenging systems in active packaging technologies.

Category of Scavenger	Mechanism	Activation Trigger	Format	Advantages	Limitations	Ref.
Iron	Iron oxidation Equations (1)–(4)	Moisture Electrolytes	Sachets Multilayer films	Fast rate Cost-effective Well-established	Moisture-dependent Potential migration Regulatory constraints Microwave incompatibility	[57,59,60,61]
Platinum group metal	Catalytic hydrogen oxidation Equation (5)	H_2_	Labels	High efficiency Rapid scavenging kinetics Significant shelf life extension	H_2_-dependent High costs	[62,63]
Ascorbic acid	Redox-mediated ascorbate oxidation Equation (6)	Moisture Transition metal traces	Sachets Films Bottles	Iron-free scavenger	High loading levels	[64]
Light	Type II photo-sensitized singlet oxygen generation Equations (7)–(9)	h*v*	Films Bottles	High absorption capacity and rate Moisture-free	Temperature Transition metal catalyst Light exposure High costs	[65,66,67,68]
Enzyme	Glucose oxidase + catalase Equations (10) and (11) Laccase Equation (12) Ethanol oxidase Oxalate Oxidase	Moisture Substrate availability	Films Coatings	Incorporation into coatings Immobilization to nanoparticles	Moisture-dependent Temperature	[69,70]
Unsaturated hydrocarbon	Autoxidation with ^3^O_2_ Equations (13)–(15)	O_2_ Heat h*v* Metal ions	Multilayer films	Moisture-free Suitable for low water activity Foods stored at room temperature	Odor-active and low molecular weight Organic acids by-products	[71,72]
Redox system	Autoxidation Equations (16)–(18)	O_2_ h*v* Heat	Multilayer films	Versatile Catalytic action Safe	Substrate activation Temperature ROS generation	[73,74]
Phenolic compound	Autoxidation Equations (19) and (20)	Alkaline environment	Films	High O_2_ absorption capacity Eco-friendly	High pH Reactive by-products	[75,76]
Microorganism	Aerobic respiration Equation (21)	Moisture	Polymer films Coatings	Eco-friendly Effective Low costs	Moisture-dependent Contamination Microorganism viability Heat lability CO_2_ production	[74,77]

H_2_ hydrogen; h*v* light (photons); ^3^O_2_ atmospheric triplet oxygen; O_2_ oxygen; CO_2_ carbon dioxide.

**Table 3:** Oxygen (O_2_) control technologies in active packaging: materials, carrier matrices, system configurations, oxygen absorption capacity and rate, functional performance, operational conditions, and food applications.

Active Material	Polymer Matrix Carrier	Technology Format	Quantitative Performance	Functional Performance	Storage Conditions	Commodity	Ref.
Iron-nanoparticles (Fe-NPs)	PET/PE	Sachet	O_2_-scavenging capacity: 134.1 ± 2.9 mL O_2_; O_2_-scavenging rate: 0.45 ± 0.044 L h^−1^	Controlled lipid oxidation; PV: 19.82 meq O_2_ kg^−1^ (sunflower seeds), 8.84 meq O_2_ kg^−1^ (walnuts); AnV: 3.83 (sunflower seeds), 1.71 (walnuts)	25 °C, 120 days	Roasted sunflower seeds; walnuts	[61]
Ageless^®^ (iron-based oxygen absorber)	-	Sachet	Headspace O_2_ reduced to <0.1 vol.%	Inhibition of mold growth and insect proliferation; reduced oxidative deterioration and discoloration	25 °C; 20 days to 6 months	Sponge cake; cereals; corn-oil; noodle; sliced ham	[98]
TOTA-PAK^®^ (iron-based oxygen absorber)	-	Sachet	Headspace O_2_ consistently reduced to <0.1 vol.%	Shelf life extension; inhibition of mold and fungal growth; preservation of color, taste, and aroma	-	Nuts; dried fruits; dehydrated snacks; tea; coffee; protein powders; meat products; biscuits; bread; spices	[99]
Iron-modified kaolinite	LLDPE	Film	O_2_-scavenging capacity: 4.3 mL O_2_ g^−1^ LLDPE	Proof-of-concept O_2_ scavenging with potential for shelf life extension of O_2_-sensitive foods	24 °C, 100% RH, 60 days	Oxygen-sensitive foods and beverages	[79]
	HDPE		O_2_-scavenging capacity: 2.4 mL O_2_ g^−1^ HDPE (100% RH); 0.7 mL O_2_ g^−1^ HDPE (50% RH, 5 °C)		24 °C/5 °C, 50–100% RH 60 days		
Pd-based catalytic system	PS–EVOH–PE	Integrated tray system	Headspace O_2_ reduced from 2 vol.% to <0.01 vol.% in 105–190 min	Mold-free shelf life extended by 3–16 days under CO_2_-MA; additional 3–9 days under O_2_ scavenger	MAP, 23 °C, 50% RH, up to 42 days	Bakery products	[100]
No-OX^®^ (iron free oxygen absorber)	Foam tray and plastic wrap	Integrated packaging system	-	Microbial growth inhibition; extended shelf life; maintenance of desirable color	-	Fresh and dried meat products	[101]
Activated carbon sodium L–ascorbate nanoparticles	PE	Sachet	Headspace O_2_ reduced from 20.9 vol.% to <1 vol.% (day 4)	34% lower TBARS vs. control; TAB, LAB, and YM growth reduced by 27.3%, 57.1%, and 85.7%, respectively	4 °C, 4 days	Raw meatloaves	[64]
Ascorbic acid	Whey protein isolate film	Film	O_2_-scavenging capacity: 35.6 ± 2.3 cc O_2_ g^−1^ dry film	Potential application for shelf life extension of O_2_-sensitive foods through headspace and permeating O_2_ scavenging	Room temperature, pH > 7, 3 days	Oxygen-sensitive foods	[85]
UV-activated	LDPE/polyisoprene	Film	O_2_-scavenging capacity: 16.72 mL O_2_ g^−1^; O_2_-scavenging rate: 2.09 mL O_2_ g^−1^ per day	Maintained color; controlled lipid oxidation (TBARS: 2.83 mg MDA kg^−1^, day 90); reduced microbial growth (TPC: 3.05 ± 0.56 CFU g^−1^, day 40)	UV activation (10 s); room temperature, 90 days	Beef jerky	[66]
UV-activated cyclo-olefin ORMOCER^®^	PE/PU multilayer laminate	Coating	O_2_-scavenging capacity: up to 242 ± 8 mg O_2_ g^−1^ of coating	Proof-of-concept O_2_ scavenger for scalable application on flexible and three-dimensional packaging formats	UV activation (6−7 J cm^−2^); 23 °C; 0% RH	Oxygen-sensitive foods	[65]
Immobilized glucose oxidase	PVA/chitosan/tea extract nanofibers	Film	Headspace O_2_ reduced from 3 vol.% to <1.1 vol.% (haw jelly) and 0.8 vol.% (cream cake); efficiency up to 73%	Efficient O_2_ removal, microbial inhibition, shelf life extension	-	Haw jelly; cream cake	[93]
Laccase + lignosulfonates	Latex-based/starch-based films	Foil/board	Headspace O_2_ reduced from 1 vol.% to <0.3 vol.% (day 6)	Proof-of-concept O_2_ scavenger for active packaging of high-moisture foods	23 °C, 100% RH	High-moisture oxygen-sensitive goods	[94]
Linoleic acid/oleic acid	Modified calcium carbonate particles (tea bags)	Sachet	O_2_-scavenging capacity: >195.6 ± 13.5 mL O_2_ g^−1^; O_2_-scavenging rate: up to 12.2 ± 0.6 O_2_ g^−1^ d^−1^	Proof-of-concept O_2_ scavenger for foods rich in unsaturated fatty acids	5 °C/30 °C, 37–100% RH	Low water activity foods; non-refrigerated	[72]
Polybutadiene	Fibrous film	Film	O_2_-scavenging capacity: 284.25 mL O_2_ g^−1^	Enhanced oxidative stability of walnut oil	63 °C (accelerated), 15 days	Walnut oil	[102]
Gallic acid	PLA/mPLA-GA film	Film strips	~17% O_2_ scavenging after 10 days	Prevented package bloating; delayed ripening; extended shelf life	Vacuum sealed; room temperature, 14 days	Banana	[103]
*K. varians*, *P. subpelliculosa*	HEC–PVOH polymeric matrices	Film	Maintained microbial viability during storage; improved O_2_ removal from package headspace	Proof-of-concept active O_2_ scavenger film for high-moisture foods	25 °C/37 °C, 50% RH, 20 days	High water activity foods	[77]

O_2_ oxygen; PET polyethylene; PE polyethylene; PV peroxide value; AnV anisidine value; LLDPE linear low-density polyethylene; HDPE high density polyethylene; RH relative humidity; Pd palladium; PS polystyrene; EVOH ethylene vinyl alcohol; MA modified atmosphere; MAP modified atmosphere packaging; TBARS thiobarbituric acid reactive substances; TAB total aerobic bacteria; LAB lactic acid bacteria; YM yeasts and molds; TPC total plate count; PU polyurethane; PVA polyvinyl alcohol; mPLA-GA multibranched PLA functionalized with gallic acid; HEC hydroxyethyl cellulose; PVOH polyvinyl alcohol.

**Table 4:** Summary of ethylene (C_2_H_4_)-scavenging and -inhibiting technologies used in active packaging systems.

Active Material	Carrier/ Format	Mechanism	Quantitative Effectiveness	Functional Performance	Storage Conditions	Commodity	Ref.
KMnO_4_ + pumice composite	LDPE + PE-g-MA film	Chemical oxidation + physical adsorption	C_2_H_4_ adsorption capacity: 7.31 μmol/film	Shelf life extended to 20 days; controlled C_2_H_4_/CO_2_ levels; reduced firmness loss	25 °C, 30% RH, 20 days	Avocado	[118]
KMnO_4_ -impregnated nanoparticles	POE nanocomposite film	Chemical oxidation	C_2_H_4_ adsorption capacity: up to 67%	No brown spots after 15 days (vs. 7 days in control); shelf life extended	Room temperature, 15 days	Banana	[122]
Gaseous O_3_ (325–350 ppm, 60 min)	-	Oxidative degradation	∼50% reduction in C_2_H_4_ respiration rate	Delayed stem-end rot; reduced disease incidence; maintained firmness and color	13 °C, 95 ± 2% RH, 20 days	Jackfruit	[125]
Gaseous O_3_ (0.15 ppm day/0.3 ppm night)	-	Oxidative degradation	~40% lower C_2_H_4_ vs. control	Slower softening; maintained firmness; reduced microbial growth	6 °C, 90% RH, 13 days	Cantaloupe melon	[127]
Aqueous ozone (1.4 mg·L^−1^)	PE cling film	Oxidative degradation	~50% lower C_2_H_4_ production vs. control	Reduced microbial load; removed pesticide residues; improved quality	14 °C, 12 days	Fresh cut cabbage	[126]
1-MCP (1 μL·L^−1^)	Plastic film	Ethylene receptor inhibition	56% lower C_2_H_4_ release vs. control (day 8)	Delayed senescence; maintained quality; preserved bioactive compounds	25 ± 1 °C, 85–90% RH, 8 days	Cabbage leaves	[131]
Al_2_SiO_5_ + Al_2_H_2_O_6_Si	-	Physical adsorption	C_2_H_4_ adsorption capacity: 84% (55% RH); 68% (75% RH)	Improved safety; extended shelf life under storage conditions	25 °C, 21 days	Fresh produce	[133]
Silver-impregnated zeolite	Chitosan-coated paper	Physical adsorption	85% lower C_2_H_4_ vs. control (day 15)	29% less weight loss; texture maintained; slower ripening vs. control	25 ± 2 °C, 30 days	Cherry tomato	[134]
Cu_2_O-modified TiO_2_ (TNT-Cu_2_O)	CNF film in PET containers	Photoinduced oxidation	50% lower residual C_2_H_4_ vs. control (160 h)	Delayed quality loss; reduced color change, weight loss, and softening	Room temperature, 14 days	Tomato	[136]
Gelatin-TiO_2_ nanocomposite	EPE foam nets	Photocatalytic oxidation	60% lower C_2_H_4_ accumulation vs. control (96 h)	Delayed ripening; reduced respiration; no scalds or fungal growth; maintained firmness and color	25–30 °C, 85% RH, 4 days	Papaya	[137]

C_2_H_4_ ethylene; KMnO_4_ magnesium permanganate; LDPE low-density polyethylene; PE-g-MA maleic anhydride-grafted polyethylene; RH relative humidity; POE polyolefin elastomer; O_3_ ozone; 1-MCP 1-methylcyclopropene; Al_2_SiO_5_ sillimanite; Al_2_H_2_O_6_Si bentonite; TNT titanium dioxide nanotubes; CNF carbon nanofibers; PET polyethylene terephthalate; EPE expanded polyethylene.

**Table 5:** Overview of carbon dioxide (CO_2_)-scavenger and -emitter systems applied in active food packaging.

Active Material	Carrier/ Format	Mechanism	Performance	Storage Conditions	Commodity	Ref.
CO_2_-emitter sachet, NaHCO_3_ + C_6_H_8_O_7_	Sachet/pad	Acid–base reaction generating CO_2_	Maintained high headspace CO_2_; prevented package collapse; limited drip loss; inhibited spoilage bacteria	4 °C	Chicken fillets	[150]
CO_2_-emitter pad	Sachet MAP/Vacuum + pad	Acid–base reaction generating CO_2_	Shelf life prolonged by 44% in MAP + CO_2_ emitter vs. MAP alone and by 29% in Vacuum + CO_2_ emitter vs. Vacuum alone	2 °C	Raw fish	[151]
NaHCO_3_ + C_6_H_8_O_7_ CO_2_-emitting sachet	PBAT-TPS/ZnO nanoparticle film based-active sachet	Acid–base reaction generating CO_2_	Regulated CO_2_ release; CO_2_ reaches ~35% headspace within 84 h; drip loss reduction	-	-	[152]
Ca(OH)_2_ + porous medium CO_2_ absorber	LLDPE sheets	Chemical absorption of CO_2_	~45% reduction in CO_2_ headspace vs. control	0 °C	Kimchi	[156]
Zeolite and/or activated carbon	Microporous Tyvek^®^ sachet	Physical adsorption of CO_2_	CO_2_ absorption and release kinetics useful to alleviate the package pressure and improve product sensory quality	0 °C/10 °C/20 °C	Kimchi	[154]
StayFresh^®^-activated carbon, calcium oxide, and sodium water glass	Sachet	Chemical adsorption of excess CO_2_	Maintains freshness and visual quality by controlling excess CO_2_ accumulation	Sealed food packaging during storage and distribution	Fresh produce/packaged foods	[158]

NaHCO_3_ sodium bicarbonate; C_6_H_8_O_7_ citric acid; MAP modified atmosphere packaging; PBAT poly(butylene adipate-co-terephthalate); TPS thermoplastic starch; ZnO zinc oxide; Ca(OH_2_) calcium hydroxide; LLDPE linear low-density polyethylene.

**Table 6:** Overview of antimicrobial packaging systems applied in food preservation.

Active Material	Polymer Matrix/Carrier	Technology Format	Functional Performance	Storage Conditions	Commodity	Ref.
Cinnamon essential oil microemulsion	Silk fibroin/chitosan	Edible antimicrobial coating	>98% inhibition against *E. coli*, *S. aureus*, *P. citrinum*, *A. niger*, *M. racemosus*, and *T. viride*; improved firmness, reduced weight loss, delayed spoilage	-	Strawberries	[205]
Clove essential oil	PET/PP film with pullulan coating	Active packaging film	Sustained antibacterial activity against *E. coli*, *S. enteritidis*, *S. aureus*, and *L. monocytogenes*; maintained visual quality	4 °C; 7–10 days	Spinach leaves	[206]
Oregano essential oil	Zein–pectin–chitosan nanoparticles	Nanoencapsulated edible coating	Strong antioxidant and antimicrobial activity; inhibited spoilage microorganisms; improved stability of bioactive compounds	20 °C; 65% RH	Red sausage	[207]
ε-Polylysine hydrochloride + nisin	Chitosan	Antimicrobial edible film	>90% inhibition against *S. aureus* and *E. coli*; improved mechanical, moisture, and oxygen barrier properties	-	Fresh peaches	[215]
ε-Poly-L-lysine + phase-transitioned lysozyme	Bio-based polymeric matrix	Rechargeable antimicrobial packaging film	Complete inactivation (~6 log CFU mL^−1^ reduction) of *S. aureus* and *E. coli* within 30 min; retained efficacy after 10 recharge cycles	-	Fresh blueberries	[216]
Zinc oxide nanoparticles (ZnO NPs)	Chitosan	Bio-nanocomposite active packaging film	Enhanced antimicrobial activity, reduced lipid oxidation, improved physicochemical properties, extended shelf life	4 ± 2 °C; 11 days	Poultry meat	[222]
Silver ion antimicrobial additive (SilverShield^®^)	Polymeric materials/food-contact plastics	Built-in antimicrobial polymer technology	Inhibition of bacterial colonization (reduction up to 99.9% of *E. coli* and *S. enteritidis*)	Ambient conditions	Food-contact materials/reusable containers	[223]
Antimold-Mild^®^ (powderized ethanol adsorbed on silica gel)	Sachet carrier (special wrapping paper/multilayer packaging)	Antimicrobial emitter sachet	Controlled ethanol vapor release to inhibit microbial growth and mold development while maintaining food softness and moisture	Ambient/controlled storage	Bakery products, confectionery, fresh produce	[224]

*E. coli Escherichia coli*; *S. aureus Staphylococcus aureus*; *P. citrinum Penicillium citrinum*; *A. niger Aspergillus niger*; *M. racemosus Mucor racemosus*; *T. viride Trichoderma viride*; *S. enteritidis Salmonella enteritidis*; *L. monocytogenes Listeria monocytogenes*; PET polyethylene terephthalate; PP polypropylene.

**Table 7:** Overview of antioxidant packaging systems applied in food preservation.

Active Material	Polymer Matrix/Carrier	Technology Format	Functional Performance	Storage Conditions	Commodity	Ref.
Grape marc/olive pomace/*Moringa oleifera* extracts	Cellulose	Active film	>50% reduction of lipid peroxidation	4 °C, 16 days	Ground beef	[236]
Rice straw extract	PLA	Biodegradable active film	Reduced oxidative spoilage	-	Food packaging	[238]
Lemon and tomato by-product extracts	PLA/GP/LDPE	Active Film	Controlled release of polyphenols; enhanced antioxidant and barrier properties	Up to 30 days	High-fat foods	[239]
Origin^®^ Powder AC 34 (acerola cherry extract)	-	Commercial antioxidant ingredient platform	Delayed metmyoglobin formation; preserved red color; improved oxidative stability	Commercial application	Meat products	[240]
Duralox^®^ Oxidation Management Systems (rosemary extract, green tea, acerola, tocopherols)	-	Clean label	Multifunctional antioxidant protection through radical scavenging, metal chelation, and oxidative stabilization; designed to replace synthetic antioxidants	Commercial application	Meat, poultry, oils, snacks, sauces, ready meals	[241]
Gallic acid-modified silica nanoparticles	Chitosan	Nanocomposite film	Strong antioxidant activity; improved mechanical strength, water vapor barrier properties, and UV barrier performance	-	Food model system	[237]
α-DL-tocopherol acetate (vitamin E)	Sodium alginate	Antioxidant edible coating	Delayed oxidative deterioration, reduced microbial spoilage, preserved physicochemical quality, extended shelf life	10 °C, 65% RH, 15 days	Strawberries	[242]

PLA poly(lactic acid); GP G-polymer; LDPE low-density polyethylene.

**Table 8:** Representative flavor and odor control agents used in food packaging applications.

System	Function	Mechanism	Examples	Applications	Ref.
Aroma/Flavor Emitters	Enhance or modify aroma/flavor attributes	Controlled release of volatile sensory-active compounds into the food/package headspace	Essential oils, ethanol emitters, encapsulated flavors, coffee aroma compounds	Bakery, coffee, fresh produce, meat, cheese, fruits	[60,253]
Aroma/Flavor Adsorbers	Remove unwanted odors/off flavors	Adsorption of volatile compounds	Activated carbon, zeolites, cyclodextrins	Fish, meat, dairy	[252]
Activated Carbon Adsorbers	Odor removal	Physical adsorption on porous surface	Carbon pads/films	Seafood, meat	[254]

**Table 9:** Representative multifunctional active packaging systems for food applications.

Active Material	Polymer Matrix/Carrier	Packaging Format	Functional Performance	Storage Conditions	Food Commodity	Ref.
Seaweed polysaccharides	Carboxymethyl chitosan	Active film	Antioxidant, antimicrobial, antiglycation, UV-blocking, barrier enhancement	Room temperature, 7 days	Strawberry	[266]
Carbon dot/g-C_3_N_4_ nanocomposite (CCN)	Corn starch/carboxymethyl cellulose (CS/CMC)	Functional film	Antioxidant, antimicrobial, lipid oxidation inhibition, shelf life extension (+4 days)	20 °C, 75% RH	Banana	[267]
Carbon dots (*Galla chinensis*-derived)	Pullulan	Active film	UV-blocking, antibacterial, antioxidant, barrier enhancement	Room temperature, 10 days	Strawberry	[268]
Cu-based nanozyme	Carrageenan	Active film	Antibacterial, enzymatic browning inhibition, antioxidant enzyme-mimetic activity	Accelerated, 35 °C	Fresh-cut apple/figs	[269]
Sodium carbonate/sodium glycinate/carvacrol	Agar-based film	Packaging insert label	Antimicrobial, moisture absorption, CO_2_ scavenging	10 °C, MAP	Shiitake mushroom	[261]
Linalool/eugenyl acetate/polyethyleneimine	β-cyclodextrin complexes	Active insert	Antimicrobial controlled release + CO_2_ scavenging	7 °C, 51% RH, up to 40 days	Cherry tomato	[270]
Zinc-based metal–organic framework (Zn-MOF) bioactives	Gelatin-based composite film	Active nanocomposite film	Antimicrobial, antioxidant, UV protection, barrier reinforcement	10 °C, up to 16 days	Tomato	[271]
Chitosan + quercetin	Chitosan	Film	Antioxidant, antimicrobial, spoilage monitoring, shape memory behavior	Accelerated, 25 °C	Fish	[262]
AGELESS^®^ O_2_ scavenger/CO_2_ generator	Commercial proprietary absorber matrix	Sachet/package insert	Oxygen scavenging, CO_2_ generation, oxidation and microbial spoilage control	Packaged storage	Bakery, meat, snacks, dried foods	[272]
Ultra Fresh^®^ enzyme-based	Proprietary enzyme/emulsifier formulation	Commercial preservation platform	Moisture retention, anti-staling, crumb softening, shelf life extension	Ambient	Bread, buns, tortillas, bakery products	[273]

## Data Availability

No new data were created or analyzed in this study. Data sharing is not applicable to this article.

## Abbreviations

The following abbreviations are used in this manuscript:

Abbreviation	Full term
1-MCP	1-Methylcyclopropene
AA	Ascorbic Acid
AnV	Anisidine Value
C_2_H_4_	Ethylene
CH_4_	Methane
CO_2_	Carbon Dioxide
DHAA	Dehydroascorbic Acid
EVOH	Ethylene Vinyl Alcohol
FAO	Food and Agriculture Organization
FLW	Food Loss and Waste
GA	Gallic Acid
GHG	Greenhouse Gas(es)
H_2_	Hydrogen
HDPE	High-Density Polyethylene
KMnO_4_	Potassium Permanganate
LAB	Lactic Acid Bacteria
LDPE	Low-Density Polyethylene
LLDPE	Linear Low-Density Polyethylene
MAP	Modified Atmosphere Packaging
MCC	Modified Calcium Carbonate
NFOSP	Nonferrous Oxygen Scavenger Particles
N_2_O	Nitrous Oxide
NRL	Natural Rubber Latex
O_2_	Oxygen
ORMOCER^®^	Organically Modified Ceramic
PBAT	Poly(butylene adipate-co-terephthalate)
PBS	Polybutylene Succinate
PBSA	Poly(butylene succinate-co-adipate)
PE	Polyethylene
PET	Polyethylene Terephthalate
PFAS	Per- and Polyfluoroalkyl Substances
PGMs	Platinum Group Metals
PLA	Polylactic Acid
PP	Polypropylene
PS	Polystyrene
PU	Polyurethane
PVA	Polyvinyl Alcohol
PV	Peroxide Value
RH	Relative Humidity
ROS	Reactive Oxygen Species
RSM	Response Surface Methodology
SDGs	Sustainable Development Goals
TBARS	Thiobarbituric Acid Reactive Substances
TiO_2_	Titanium Dioxide
TPS	Thermoplastic Starch
UFAs	Unsaturated Fatty Acids
UV	Ultraviolet
UV-C	Ultraviolet C
WFP	World Food Programme
